# Histone acetylation in an Alzheimer’s disease cell model promotes homeostatic amyloid-reducing pathways

**DOI:** 10.1186/s40478-023-01696-6

**Published:** 2024-01-02

**Authors:** Daniel C. Xu, Hanna Sas-Nowosielska, Greg Donahue, Hua Huang, Naemeh Pourshafie, Charly R. Good, Shelley L. Berger

**Affiliations:** Department of Cell and Developmental Biology, Perelman School of Medicine Philadelphia, Penn Institute of Epigenetics, Philadelphia, PA 19104 USA

## Abstract

Alzheimer’s Disease (AD) is a disorder characterized by cognitive decline, neurodegeneration, and accumulation of amyloid plaques and tau neurofibrillary tangles in the brain. Dysregulation of epigenetic histone modifications may lead to expression of transcriptional programs that play a role either in protecting against disease genesis or in worsening of disease pathology. One such histone modification, acetylation of histone H3 lysine residue 27 (H3K27ac), is primarily localized to genomic enhancer regions and promotes active gene transcription. We previously discovered H3K27ac to be more abundant in AD patient brain tissue compared to the brains of age-matched non-demented controls. In this study, we use iPSC-neurons derived from familial AD patients with an amyloid precursor protein (APP) duplication (APP^Dup^ neurons) as a model to study the functional effect of lowering CBP/P300 enzymes that catalyze H3K27ac. We found that homeostatic amyloid-reducing genes were upregulated in the APP^Dup^ neurons compared to non-demented controls. We lowered CBP/P300 to reduce H3K27ac, which led to decreased expression of numerous of these homeostatic amyloid-reducing genes, along with increased extracellular secretion of a toxic amyloid-β species, Aβ(1–42). Our findings suggest that epigenomic histone acetylation, including H3K27ac, drives expression of compensatory genetic programs in response to AD-associated insults, specifically those resulting from APP duplication, and thus may play a role in mitigating AD pathology in neurons.

**Supplementary Information:**

The online version contains supplementary material available at 10.1186/s40478-023-01696-6.

## Introduction

Alzheimer’s Disease (AD), the most common form of senile dementia, is a neurodegenerative disease characterized clinically by irreversible memory loss and cognitive decline [1]. The pathology of AD is comprised mainly of amyloid-β (Aβ) plaques [2–5] and tau neurofibrillary tangles [6]. However, the relationship of these pathological hallmarks to each other and to disease features such as cognitive decline and neurodegeneration remain incompletely understood [7, 8].

Advances in understanding of chromatin architecture dynamics during normal aging and during neurodegeneration have revealed epigenomic dysregulation as a potential important link connecting aging, disease pathological features, and neurodegeneration [9]. In one example, we and others have uncovered a disease-association for histone 3 acetylation at lysine residue 27 (H3K27ac), a histone modification catalyzed by the lysine acetyltransferases p300 (EP300) and CBP (CREBBP) [10, 11]. H3K27ac is found primarily in enhancer regions, and promotes active transcription of genes [12–14]. However, the balance of epigenomic H3K27ac gains and losses (genomic peaks measured using ChIPseq) is less clear, and indeed may be brain region-dependent and gene-dependent. Specifically, while we observed a substantial net increase in the number of H3K27ac peaks in the lateral temporal lobe of AD patients [15], there was a net decrease in H3K27ac peaks in the entorhinal cortex (although a substantial number of H3K27ac peaks were still gained in AD patient samples compared to control) [16]. Hence, the precise occurrence and role of enhancer-associated H3K27ac may be complex and gene-specific, and requires additional clarification.

Efforts to determine a functional relationship, whether disease-promoting or disease-ameliorating, between altered H3K27ac abundance and pathology are complicated by the complexity of brains and the difficulty in establishing causality in postmortem tissue samples, where pathology and epigenomic dysregulation are co-occurrent. We therefore sought to investigate these questions in an iPSC-neuron model. Methods for induction of cortical neurons from iPSCs (iPSC-neurons) through overexpression of the master neuronal transcriptional regulator NGN2 have been well-established to produce excitatory cortical neuronal cells with high speed, consistency, and efficiency [17–19], and iPSC-neurons are increasingly utilized as a tractable model for neurodegenerative diseases [19–22]. We leveraged these properties to generate direct epigenomic perturbations in a homogenous population of neurons, and to examine downstream effects on gene expression and AD-related pathology.

Here, we performed transcriptomic and functional characterization of iPSC-neurons derived from non-demented control (NDC) donors and from familial Alzheimer’s Disease patient donors harboring an amyloid precursor protein (APP) duplication (APP^Dup^). Our results provide additional evidence for the important role played by chromatin-regulating histone acetylation in neurodegenerative disease, and reveal a homeostatic protective mechanism whereby neurons regulate gene transcription in response to AD pathology.

## Results

### Generation of forebrain-like cortical neurons from APP^Dup^ and NDC iPSCs

We obtained two APP duplication (APP^Dup^) and two non-demented control (NDC) iPSC lines previously established as a model of familial AD [21]. We validated via genomic qPCR that both APP^Dup^ iPSC lines maintained 3 copies of APP compared to 2 copies maintained by both NDC lines (Additional file 2: Fig. S1A). These cell lines were previously induced using a small-molecule-based neuron induction system and shown to exhibit differences in amyloid and tau species generation upon differentiation [21]. Importantly, the genome and epigenome of these cells have not been explored. Here, we utilized a single-step genetic Ngn2 overexpression strategy to differentiate these lines robustly and consistently to facilitate whole-transcriptome analysis.

**Fig. 1:**
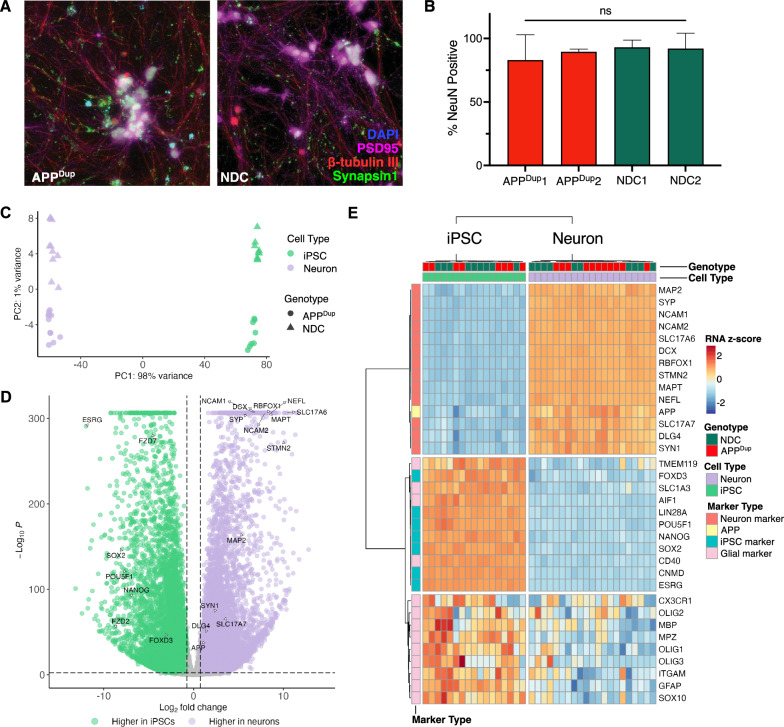
APPDup and NDC iPSC lines differentiate successfully into excitatory cortical neurons. **A** Representative immunofluorescence staining for DAPI, beta tubulin III, NeuN and synapsin1 in APPDup and NDC neurons day 21 CTRL RNAi-treated neurons. **B** No significant differences in quantification NeuN + % nuclei were observed among APPDup and NDC differentiated neurons (*p* = 0.814, one-way ANOVA). Error bars indicate s.e.m. **C** Principal Component Analysis (PCA) plot of variation of top RNA-Seq genes from differentiated neurons and iPSCs from all cell lines. **D** Volcano plot depicting differentially expressed genes (DEGs) between APPDup and NDC CTRL RNAi-treated day 21 neurons and iPSCs. Significance and foldchange threshold of p-adj. < 0.005 and log2FC > 0.75 were used. Max -log10(*p*) value plotted = 300. **E** Heatmap representing selected neuronal, iPSC, and glial marker gene expression in APPDup and NDC day 21 neurons and iPSCs

We introduced cassettes for Tet-On doxycycline-inducible promoter-driven human NGN2, and for EF1α constitutive promoter-driven mApple and blasticidin resistance, into the CLYBL safe harbor locus of each cell line via an established CRISPR-Cas9 transfection strategy [17, 18, 23] **(**Additional file 2: Fig. S1B). Transfected cells were sequentially selected for blasticidin resistance and mApple positivity to establish stable dox-inducible NGN2 transgenic lines. Following established protocols [17, 18], we induced with doxycycline and transitioned to neuronal culture media to direct differentiation of iPSCs into excitatory forebrain-like cortical neurons. Rapid induction of iPSC-neurons through forced overexpression of NGN2 yields functional neurons as measured by electrophysiology recordings by day 21 of neuronal differentiation [17, 18, 24], and thus we harvested neurons at day 21 for our experiments. We confirmed mature cortical neuronal identity by immunofluorescence (IF) staining for β-tubulin III, Synapsin1,and PSD95 **(**Fig. 1A**, **Additional file 2: Fig. S1C, D). We also stained for nuclear NeuN and observed no difference in quantified nuclear NeuN positivity between differentiated APP^Dup^ and NDC neurons (Fig. 1B).

We used RNA-seq to identify transcriptomic changes between iPSCs and 21-day neurons, and consequences of the APP duplication. We observed consistent, broad transcriptional differences between undifferentiated iPSC and differentiated neurons using principal component analysis of differentially expressed genes (DEGs) (Fig. 1C). We observed 12,496 genes upregulated in differentiated neurons passing a log2FC threshold of > 0.75 and p-adj. threshold of < 0.005, including neuron associated genes SYN1, MAPT, PSD95 (DLG4), and VGLUT1 (SLC17A7), contrasting with 7398 genes upregulated in iPSCs, including the iPSC-associated pluripotency genes OCT4 (POU5F1), SOX2, ESRG, and NANOG [25, 26]** (**Fig. 1D). We used the TissueEnrich R package to examine the most significantly upregulated set of genes following induction into differentiated neurons, and observed that the category with the highest fold-change **(**Additional file 2: Fig. S1E) and significance **(**Additional file 2: Fig. S1F) by tissue enrichment were cerebral cortex-associated genes**.** Additionally, unsupervised hierarchical clustering of selected neuronal, iPSC, and glial marker gene expression in our RNA-seq data revealed robust and distinct clusters made up of differentiated neurons and iPSCs clusters **(**Fig. 1E**)**. We observed robust upregulation of excitatory cortical neuronal marker genes across all differentiated cell lines, and downregulation of iPSC glial marker genes **(**Fig. 1E**)**. As expected, no difference in expression of cell type markers was observed between neurons derived from the APP^Dup^ iPSC and NDC iPSC lines **(**Fig. 1E**, **Additional file 1: Table S1). Thus, all iPSC lines successfully differentiated into excitatory cortical neurons, and APP duplication status did not alter neuronal differentiation.

### APP^Dup^ and NDC neurons differ in gene expression and Aβ42 secretion

**Fig. 2:**
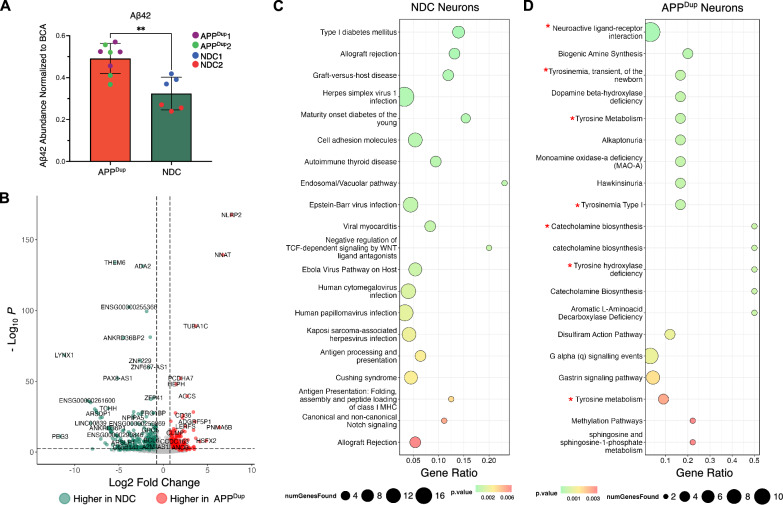
Differences between APPDup and NDC neurons. **A** APPDup day 21 neurons secrete more Aβ42 than NDC day 21 neurons (*p* = 0.0013, two-tailed T-test) at baseline. Media from CTRL RNAi-treated day 21 neurons was collected and measured via enzyme-linked immunosorbent assay (ELISA) and measured Aβ42 abundance was normalized to BCA. **B** Volcano plot depicting DEGs between APPDup and NDC day 21 neurons. Significance and fold-change threshold of p-adj. < 0.005 and log2FC > 0.75 were used. **C**, **D** Bubble plots depicting the top 25 ConsensusPathDB-identified over-represented terms in NDC-neuronoverexpressed (**C**) and APPDup neuron-overexpressed (**D**) genes. Neuron-related and tyrosine metabolismrelated terms are highlighted with an asterisk

Β-amyloid(1–42) (Aβ42) is the dominant peptide found in amyloid plaques in AD patients [3]. Thus, we examined Aβ42 in the AD patient-derived APP^Dup^ neurons using an enzyme-linked immunosorbent assay (ELISA) on neuronal culture media harvested on day 21 of differentiation. Compared to NDC controls, the media from APP^Dup^ neurons contained a significantly higher abundance of Aβ42 (Fig. 2A**)**, demonstrating an expected negative consequence of APP duplication.

Next, we determined how the transcriptomes of APP^Dup^ derived cells differ from NDC cells at both the iPSC and day 21 neuron stage. We found 1,355 genes upregulated in APP^Dup^ and 593 genes upregulated in NDC iPSCs passing a threshold of log-twofold-change > 0.75 and *p*-value < 0.005 **(**Additional file 2: Fig. S2A). At day 21 of differentiation, compared to NDC neurons, 175 genes were upregulated in APP^Dup^ neurons and 332 genes were downregulated (Fig. 2B**)**. Plotting the whole-transcriptome gene expression changes in APP^Dup^ vs NDC neurons against the whole-transcriptome gene expression changes in APP^Dup^ vs NDC iPSCs, we observed only a mild correlation of gene expression changes (R = 0.14, Additional file 2: Fig. S2B), establishing that APP^Dup^ affects gene expression differently in iPSCs than in differentiated neurons, and thus the effects of APP^Dup^ on gene expression are context-dependent.

At the iPSC stage, ConsensusPathDB pathway analysis [27, 28] uncovered no significant enrichment of neuron-related terms among significantly upregulated genes in either NDC nor APP^Dup^ cells **(**Additional file 2: Fig. S2C, D). However, neurotransmitter clearance and neuroactive ligand-receptor interaction terms were significantly enriched in APP^Dup^ neurons vs. NDC neurons (Fig. 2C, D). In addition, tyrosine metabolism pathways and monoamine oxidase-a (MAO-A) deficiency pathways were enriched in APP^Dup^ neurons (Fig. 2C, D)—consistent with findings that serum tyrosine is decreased in AD [29], and that MAO-A catalyzes the cleavage of APP into amyloid species [30]. Of note, we observed that immune processes, especially responses to viral infections, were enriched in NDC neurons compared to APP^Dup^ neurons **(**Fig. 2C**)**, which likely reflects characteristics specific to the original donors, though immune responses to HSV-1 [31] and EBV [32] have been linked to AD pathophysiology. Importantly, many genes upregulated in APP^Dup^ neurons (red) or downregulated (blue) compared to NDC neurons were identified as involved in the Alzheimer’s Disease Pathway in the Kyoto Encyclopedia of Genes and Genomes (KEGG) [33] (Additional file 2: Fig. S3). Together, these findings suggest that the underlying baseline transcriptional differences between APP^Dup^ vs. NDC cells are dependent on cell type, and are consistent with known AD-associated pathways.

### EP300/CBP knockdown mediates widespread reduction in gene transcription

**Fig. 3:**
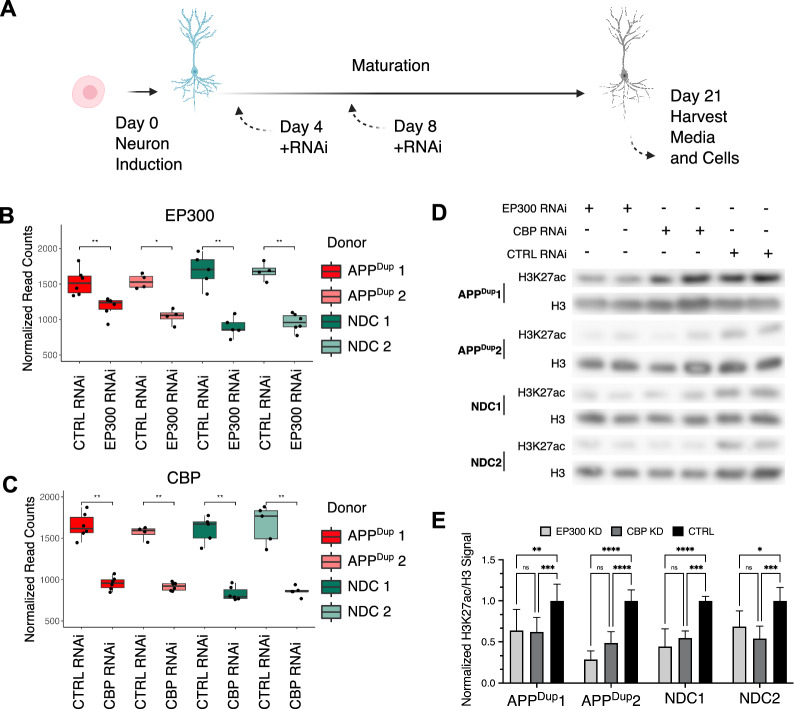
EP300/CBP knockdown leads to reduced H3K27ac in APPDup and NDC neurons. **A** Schematic detailing RNAi knockdown during iPSC-neurons differentiation. Cells were transfected with siRNA constructs at Day 4 of neuronal induction and again on Day 8; cells and media for genetic and molecular assays were harvested on day 21. B, **C** siRNA knockdown of CBP (**B**) and EP300 (**C**) results in reduction of respective transcripts lasting until day 21 (p < 0.05 for all comparisons, unpaired two-tailed Ttest). Normalized read-counts were obtained by RNA-Seq. **D** CBP/EP300 KD result in decreased acetylation of H3K27 in both APPDup and NDC neurons at day 21. Representative Western blots of protein levels of H3K27ac and total H3. **E** Quantification of H3K27ac/H3 signal across three independent iPSCneuron differentiations and RNAi treatments

In humans, H3K27 is primarily acetylated by the HATs EP300 and CBP [10, 34, 35], and is one histone modification of regulatory enhancers and promoters. Therefore, to reduce H3K27ac in our iPSC-neuron model, we employed short interfering RNA (siRNA) to knock down expression of EP300 or CBP in iPSC-neurons (Fig. 3A). Of note, EP300 and CBP are equally expressed in NDC and APP^Dup^ neurons, and no significant difference in expression was observed (Additional file 2: Fig. S4A,B). To avoid the potential interference of EP300/CBP depletion in neuronal differentiation, siRNA treatment was initiated on day 4 of the neuronal induction protocol, at which time robust neuronal morphology begins to manifest [17, 18], and then repeated at day 8 (Fig. 3A).

We analyzed RNAseq levels of EP300 and CBP in treated day 21 neurons, confirming that EP300 and CBP knockdown (KD) was maintained until day 21 of neuronal differentiation (Fig. 3B, C), and corroborated previous reports that RNAi KD persists for weeks in post-mitotic cell types [36]. To determine the effect of EP300 and CBP KD on the overall abundance of H3K27ac, we performed western blot for H3K27ac with overall histone H3 as a loading control. Commensurate with EP300 and CBP transcriptional decrease, H3K27ac was also decreased at day 21 of neuronal differentiation in all cell lines (Fig. 3D**,** quantified in Fig. 3E).

**Fig. 4:**
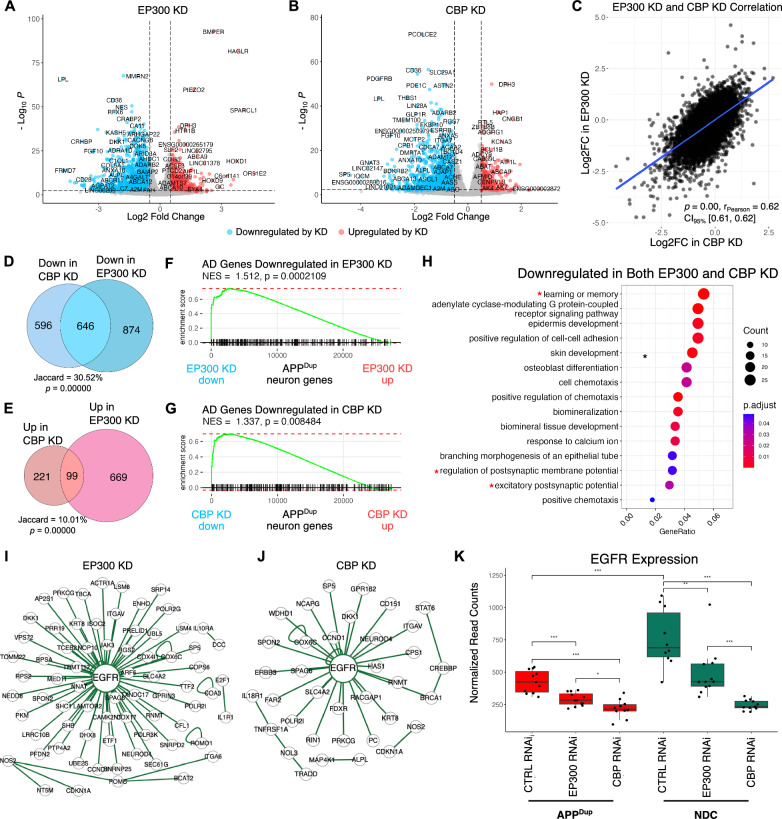
Transcriptional effects of EP300/CBP KD in APPDup neurons. **A**, **B** EP300/CBP KD results in widespread transcriptional downregulation in APPDup neurons. Volcano plot of gene expression changes between EP300 KD vs CTRL siRNA (**A**) and CBP KD vs CTRL siRNA (**B**) in APPDup neurons. Significance and fold-change threshold of p-adj. < 0.005 and log2FC > 0.50 were used. **C** EP300 and CBP KD evoke generally similar transcriptional responses. Scatterplot depicting relationship of gene transcriptional changes between EP300 KD and CBP KD (rpearson = 0.62, p = 0.00) in APPDup neurons. **D**,**E** Venn diagram showing overlaps of gene expression changes between EP300 and CBP KD in APPDup neurons. Jaccard index = 646/2116 genes, p = 0.00000, hypergeometric test for overlaps between downregulated genes (**D**). Jaccard index = 99/989 genes; p = 0.00000, hypergeometric test for overlaps between upregulated genes (**E**). **F**, **G** KEGG-identified AD-associated genes are positively transcriptionally controlled by EP300 (**F**) and CBP (**G**) in APPDup neurons. Gene set enrichment analysis (GSEA) plot depicting placement of KEGG AD genes in transcriptome ranked from highest expressed in CTRL RNAi to highest expressed in EP300 (**F**) and CBP (**E**) KD in APPDup neurons. **H** Bubble plot depicting Gene Ontology (GO) terms identified by ClusterProfiler as significantly enriched in genes downregulated in both EP300 and CBP KD in APPDup neurons. Neuron-related terms are asterisked. **I**,**J** EGFR is a central interactor of genes significantly downregulated by EP300 and CBP KD in APPDup neurons. BioGRID-identified genetic interactions depicted by EasyNetworks (esyN) of genes significantly downregulated by EP300 (**I**) and CBP (**J**) KD. **K** EGFR transcription is higher in NDC neurons at baseline (+ CTRL RNAi) and is decreased by EP300/CBP KD in both APPDup and NDC neurons (*p* < 0.05 for all comparisons, unpaired two-tailed T-test). Normalized read-counts were obtained by RNA-Seq

We examined whole-transcriptome gene expression changes via RNAseq following EP300 and CBP KD in APP^Dup^ and NDC day 21 neurons compared to siRNA controls. Upon KD of either HAT enzyme, we observed widespread gene transcription changes in both cell lines that met robustness and significance criteria of log-twofold-change > 0.50 and *p*-value < 0.005 (Fig. 4A, B). In total, in APP^Dup^ neurons, 1520 genes were downregulated in EP300 (Fig. 4A) and 1242 genes were downregulated in CBP KD (Fig. 4B); in NDC neurons, 1123 and 741 genes were downregulated in EP300 and CBP KD, respectively (Additional file 2: Fig. S5A, B). Although EP300/CBP and H3K27ac are primarily associated with enhancer regions and actively transcribed genes [12–14], we also observed the upregulation of some genes in KD conditions, which may result from secondary effects. Specifically, 768 and 320 genes were upregulated upon EP300 and CBP KD, respectively, in APP^Dup^ neurons (Fig. 4A, B), and 566 and 250 genes were upregulated upon EP300 and CBP KD, respectively, in NDC neurons (Additional file 2: Fig. S5A, B). In summary, we observed anticipated widespread downregulation of gene transcription upon KD of either EP300 or CBP, and the number of affected genes was slightly, but consistently greater in APP^Dup^ neurons compared to NDC neurons. To confirm that HAT KD did not grossly interfere with neuronal differentiation, we analyzed the expression patterns of the panel of neuronal markers we used in Fig. 1E and discovered that only one neuronal marker, SLC17A7, was significantly downregulated upon CBP KD in both APP^Dup^ and NDC neurons (Additional file 1: Tables 3, 5). No other neuronal marker crossed the significance threshold of *p-adj.* < 0.05 and absolute log2 fold-change > 0.05 in either knockout condition, in either cell line (Additional file 1: Tables 2–5).

To determine whether EP300 and CBP regulate a similar set of genes, we plotted gene expression changes in EP300 KD vs CBP KD relative to siRNA controls. We observed a strong, significant correlation in whole-transcriptomic RNA expression in EP300 and CBP KD in both APP^Dup^ (Fig. 4C) and NDC neurons (Additional file 2: Fig. S5C). We examined only the most significantly changed genes in KD conditions in APP^Dup^ neurons: the downregulated genes in EP300 and CBP KD showed a high degree of overlap (Jaccard = 30.5%, p = 0.00000) (Fig. 4D); in contrast, the upregulated genes showed a much smaller overlap (Fig. 4E), although the overlap did pass hypergeometric significance testing (Jaccard = 10%; p = 0.00000). A similar degree of overlap was observed in NDC neurons (Additional file 2: S5D, E). Taken together, these data support the view that KD of either EP300 or CBP results in broadly similar effects on overall transcription, with positive gene regulation (i.e. downregulation in enzyme KD) displaying both greater magnitude and similarity between the acetyltransferases compared to negative regulation (i.e. upregulation in enzyme KD).

### Reduction of EP300/CBP affects neuropeptide signaling pathway genes and Alzheimer’s disease pathway genes

We investigated whether EP300 and CBP KD affects genes related to AD, using Gene Set Enrichment Analysis (GSEA) on AD pathway genes identified by KEGG. GSEA revealed that genes downregulated in EP300 and CBP KD were strongly enriched for AD pathway genes in both APP^Dup^ (Fig. 4F, 4G) and NDC neurons (Additional file 2: S5F, G), indicating that EP300 and CBP drive expression of AD-related genes regardless of APP duplication status. Though AD pathway genes are not significantly enriched in APP^Dup^ neurons relative to NDC neurons (Additional file 2: S6A), a significant overlap exists between the set of genes upregulated in APP^Dup^ relative to NDC neurons and the sets of genes downregulated in HAT knockdown conditions in APP^Dup^ neurons (Additional file 2: Fig. S6B), suggesting the involvement of H3K27ac HAT enzymes in transcriptional changes brought about by APP duplication. Finally, KEGG pathway analysis revealed that the majority of the AD-related DEGs in both EP300 (Additional file 2: Fig. S7) and CBP KD (Additional file 2: Fig. S8) in APP^Dup^ neurons are downregulated (blue), supporting the view that EP300 and CBP positively regulate the expression of AD-related genes.

We performed Gene Ontology (GO) analysis [37–39] on the set of 646 genes downregulated in both EP300 and CBP KD in APP^Dup^ neurons and found that “learning and memory” was the most significantly enriched biological process (BP) GO term with the highest number of genes represented (Fig. 4H). Also enriched were neuron-related GO terms, such as “regulation of postsynaptic membrane potential” and “excitatory postsynaptic potential” (Fig. 4H). These findings reflect the known molecular role of EP300 and CBP in learning and memory and neuronal function [40, 41]. In NDC neurons, within the set of 453 genes downregulated in both EP300 and CBP KD, again “learning and memory” was the most significantly enriched GO term, along with neuron-related GO terms (Additional file 2: Fig. S9A). We performed Cell Compartment (CC) GO analysis on both sets of genes to assess the cellular location of action of EP300- and CBP-controlled genes, and discovered that the “neuron projection terminus” and “axon terminus” GO terms were among the highest enriched GO terms in both APP^Dup^ (Additional file 2: Fig. S9B) and NDC neurons (Additional file 2: Fig S9C). Together, these results confirm that the genes downregulated in EP300 and CBP KD in both APP^Dup^ and NDC neurons are enriched for neuron-related activity.

We performed Easy Networks analysis (eSyn) [42] to determine genetic associations among the EP300/CBP-regulated gene sets in the biological General Repository for Interaction Datasets (BioGRID) [43]. This analysis uncovered the epidermal growth factor receptor (EGFR) as highly central to the interactions among the genes significantly downregulated in both EP300 KD (Fig. 4I, Additional file 2: Fig. S9D) and CBP KD (Fig. 4J, Additional file 2: Fig. S9E) in both APP^Dup^ (Fig. 4I, J) and NDC neurons (Additional file 2: Fig. S9D, E). EGFR interacts with Aβ42 to synergistically promote memory loss in *Drosophila* AD models [44, 45], and is increased in an APP/PS1 transgenic AD mouse model [46]. We examined EGFR expression in EP300/CBP KD, and found that KD in both APP^Dup^ and NDC neurons significantly reduced EGFR expression (Fig. 4K). We noted lower baseline expression of EGFR in APP^Dup^ neurons compared to NDC neurons (Fig. 4K), which we discuss below. These findings show central involvement of EGFR in EP300 and CBP KD-mediated gene expression changes in both APP^Dup^ and NDC neurons.

### EP300/CBP-regulated genes are more highly expressed in the transcriptomes of healthy, non-demented aged brains compared to sporadic AD patient brains

In our previous studies of histone acetylation in human postmortem brain, we detected condition-dependent H3K27 acetylation at genomic regions specific to either AD or healthy aging, with a net increase in H3K27ac observed in AD compared to aged normal donors (“Old”) [15]. We also observed a correlation between H3K27ac ChIPseq peak enrichment and RNA-seq gene expression [15]. We therefore investigated whether the EP300/CBP regulated genes share similarity with gene expression differences between AD patient and Old brains.

**Fig. 5:**
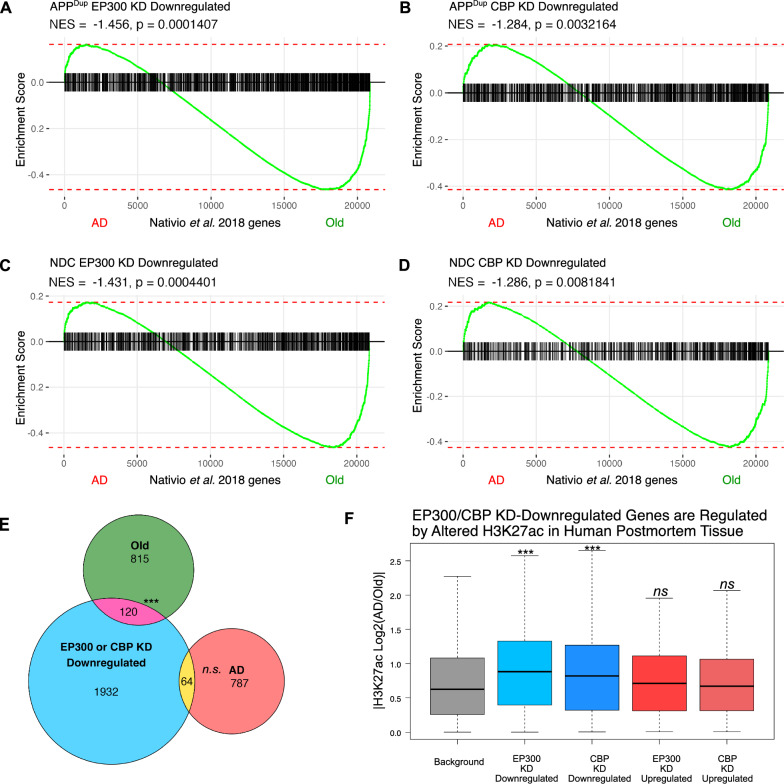
EP300/CBP-regulated genes are affected by AD-associated epigenomic dysregulation. **A**–**D** GSEA plots depicting the placement of genes significantly downregulated by EP300 KD in APPDup neurons (**A**), genes significantly downregulated by CBP KD in APPDup neurons (**B**), genes significantly downregulated by EP300 KD in NDC neurons (**C**), and genes significantly downregulated by CBP KD in NDC neurons (**D**), identified in this study, within the RNA-seq transcriptome of human whole brain, ranked from highest expressed in AD patient brains to highest expressed in non-demented age-matched (“Old”) brains from Nativio et al. 2018. **E** Significant overlap was observed between APPDup EP300/CBP KDDownregulated genes and Old-associated genes (N = 120, Jaccard = 120/3718, *p* = 2.61e-08), but not between and APPDup EP300/CBP KD-downregulated genes and AD-associated genes (N = 64, Jaccard = 64/3718, *p* = 0.613). Hypergeometric statistical testing was performed using the SuperExactTest R package. **F** EP300- and CBP-regulated genes in APPDup neurons are significantly associated with either AD-specific or Old-specific H3K27ac enrichment in human postmortem tissue, when contrasted with a control group of HAT-insensitive background genes that have nearby H3K27ac enrichment in the same brains. Statistical significance calculated using permutation test

Gene Set Enrichment Analysis (GSEA) revealed that genes downregulated in EP300 and CBP KD (i.e. CBP- and EP300-regulated in our system) were more highly expressed in Old brains in both APP^Dup^ (Fig. 5A, B) and NDC (Fig. 5C, D) neurons; we note that a fraction of significant EP300- and CBP-positively regulated genes were highly expressed in AD patients (Fig. 5A–D). We observed a slight but significant overlap between genes downregulated in either EP300 or CBP KD in APP^Dup^ neurons and genes upregulated in Old brain compared to AD brains (Jaccard index = 120/3718 genes, *p* = 2.61e-08), but no significant overlap between EP300/CBP KD-downregulated genes and AD-upregulated genes (Jaccard index = 64/3718, *p* = 0.613) (Fig. 5E). We observed the same pattern in NDC neurons, where EP300/CBP KD-downregulated genes overlapped significantly with Old-upregulated genes (Jaccard index = 80/2226, p = 6.65e-06) but not with AD-upregulated genes (Jaccard index = 40/2142, p = 0.75) (Additional file 2: Fig. S10A). In both APP^Dup^ and NDC neurons, the set of genes overlapping between EP300/CBP KD downregulated and Old-upregulated genes were most significantly enriched for neuronal processes (Additional file 2: Fig. S10B, C). Thus, EP300/CBP-regulated genes more closely overlap with a healthy aged transcriptional signature than with a diseased transcriptional signature, and this overlap is most significantly enriched for neuron-related processes.

We then examined whether the association between in vitro EP300/CBP KD and human brain gene expression is correlated with the acetyltransferase behavior of these enzymes in vivo*.* To do this, we examined whether genes positively regulated by EP300 and CBP in APP^Dup^ neurons are subject to lability of H3K27ac regulation in human brain, as measured by the previously published ChIP-seq datasets. We found that the genes downregulated in CBP/EP300 KD in both APP^Dup^ (Fig. 5F, blue) and NDC neurons (Additional file 2: Fig. S11A, blue bars) were significantly associated with altered H3K27ac enrichment in either AD patient or Old brains; these were compared to upregulated genes (Fig. 5F, Additional file 2: Fig. S11A, red bars) or background genes unaffected by CBP/EP300 KD (grey bars). Additionally, the total number of H3K27ac associated peaks per gene in human brain tissue were significantly increased over background for genes downregulated in CBP/ EP300 KD in both APP^Dup^ and NDC neurons (Additional file 2: Fig. S11B); these data suggest that genes regulated by CBP and EP300 in differentiated neurons are subject to more complex regulation in the human brain than expected by chance. In summary, CBP/EP300 regulated genes in APP^Dup^ and NDC neurons are highly expressed in Old brain, and these genes have differential, complex H3K27ac regulation between AD patient and Old brains.

### Reduction of EP300/CBP downregulates amyloid-reducing genes and increase secretion of amyloid-β(1–42)

**Fig. 6:**
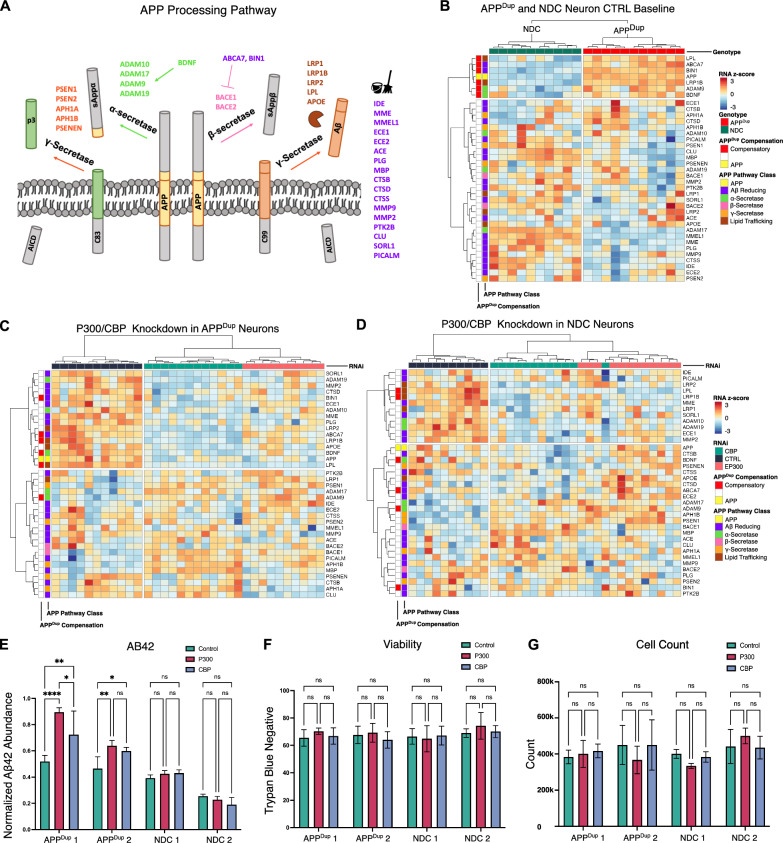
EP300/CBP KD results in downregulation of amyloid-reducing compensatory pathway in APPDup neurons. **A** Schematic of analyzed APP processing pathway genes and their functions within the APP processing pathway. **B** Amyloid-β reduction and α-secretase pathway activation genes are upregulated in APPDup vs. NDC neurons. Heatmap comparing gene expression of APP processing pathway genes across all APPDup and NDC CTRL RNAi-treated neurons. **C**, **D** Amyloid-reducing genes including those upregulated in APPDup neurons are downregulated upon EP300/CBP KD in APPDup neurons (**C**). Fewer amyloid-reducing genes are downregulated upon EP300/CBP KD in NDC neurons (**D**). **E** Aβ42 secretion is increased upon EP300/CBP KD in APPDup but not NDC neurons. Media from EP300, CBP, and CTRL siRNA-treated day 21 neurons was collected and measured via ELISA and measured Aβ42 abundance was normalized to total media protein measured by BCA. **F**, **G** EP300/CBP KD does not affect neuron viability or cell count. Day 21 neurons were collected and stained with trypan blue and under counted via hemocytometer microscopy; trypan-blue negativity (**F**) and trypan-blue negative cell count (**G**) were recorded to determine viability and cell count number

Given the APP gene duplication, we investigated whether APP pathway-specific genes were differentially expressed between APP^Dup^ and NDC neurons, and if so, whether EP300 and CBP KD alter this. We curated a list of 37 genes involved in APP processing into Aβ in humans (Fig. 6A). These include genes expected to increase amyloid abundance, such as members of the β-secretase, and γ-secretase complexes. We also included, with no a priori knowledge of expression pattern in the described model, genes identified by literature review to contribute to amyloid reduction, either through clearance or decreased production, such as the members of the α-secretase complex and other genes regulating production of amyloid-β species from APP or clearance of amyloid-β (Fig. 6A).

First, we examined whether expression of the APP-Aβ pathway-associated genes differed between APP^Dup^ and NDC neurons. We performed hierarchical clustering on the normalized RNA-seq expression data in APP^Dup^ and NDC neurons and identified, in addition to APP itself, a cluster of 6 APP pathway-associated genes that were uniformly more highly expressed in APP^Dup^ neurons compared to NDC neurons: *LPL*, *ABCA7*, *BIN1*, *LRP1B*, *ADAM9*, and *BDNF* (Fig. 6B, top group outlined in red on left). These genes are implicated to reduce amyloid, suggesting that their upregulation in the APP^Dup^ neurons represents a homeostatic, compensatory response to reduce the level of Aβ.

Next, we examined whether EP300 or CBP reduction affects the expression of these and other amyloid pathway genes in APP^Dup^ neurons. We found a cluster of 16 genes downregulated in EP300/CBP KD conditions compared to control siRNA treatment, including 5/6 of the compensatory pathway genes found to be upregulated in APP^Dup^ vs NDC neurons: BIN1, ABCA7, LRP1B, BDNF, and LPL (Fig. 6C, outlined in red on left). In addition to BDNF, which promotes α-secretase activity and inhibits β-secretase activity [47, 48], the α-secretase complex members ADAM19 and ADAM10 were also decreased upon EP300/CBP KD. The α-secretase complex diverts APP substrate toward α- rather than β-cleavage, reducing Aβ production [49]. ABCA7 [50, 51] and BIN1 [52–54] were identified as APP^Dup^ compensatory and also identified as decreased in EP300/CBP KD; these genes reduce amyloid-β by negatively regulating the activity of β-secretase complex member BACE1 [50–53]. Additionally, EP300/CBP KD resulted in the decreased transcription of SORL1 [55], MMP2,CTSD [57], ECE1, MME [59, 60], and PLG which reduce Aβ through amyloid catabolism. We note that some Aβ reducing genes were not downregulated upon KD, though many of these genes, such as MMEL1, IDE, ECE2, and MMP9 appear to be more lowly expressed at baseline in APP^Dup^ cells compared to NDC cells (Fig. 6B). Finally, the lipid trafficking and metabolism proteins APOE, LPL and LRP family members LRP1B and LRP2, which mediate amyloid reduction through co-aggregation with amyloid species and prevention of their extracellular aggregation [22, 63–67], were reduced upon EP300/CBP KD; of these, LPL and LRP1B were also identified as APP^Dup^-compensatory. These findings indicate that knockdown of EP300/CBP tended to decrease expression of homeostatic genes that maintain low levels of toxic Aβ.

Interestingly, the transcriptional response to EP300/CBP KD in NDC neurons differed from APP^Dup^ neurons. Select APP^Dup^-compensatory genes that were decreased by EP300/CBP KD in APP^Dup^ neurons (ABCA7, BIN1, BDNF), were not decreased by EP300/CBP KD in NDC neurons (Fig. 6D). NDC neurons still showed a cluster of downregulated amyloid-reducing genes upon EP300/CBP KD; however, the total number of affected genes was smaller (12 genes compared to 16 genes in APP^Dup^ neurons), and included only 2/6 of the APP^Dup^ neuron-upregulated compensatory genes: LPL and LRP1B. Hence, genes in the APP-Aβ pathway, particularly those involved in Aβ reduction, were decreasing in EP300/CBP KD, dependent on underlying genetic background of the neurons, with broader downregulation in APP^Dup^ neurons compared to NDC neurons.

While expression of APP itself was increased, as expected, in APP^Dup^ neurons compared to NDC neurons (Fig. 6B), expression of APP decreased upon EP300/CBP KD in both APP^Dup^ and NDC neurons (Fig. 6C, D). It is possible that feedback sensing of a decreased APP abundance induced by EP300/CBP KD contributed to the downregulation of amyloid-reducing genes, mirroring the induction of amyloid-reducing compensatory genes in APP^Dup^ neurons. Additionally, the Aβ-producing β-secretase gene BACE1 was significantly upregulated in both EP300 and CBP KD in APP^Dup^ neurons, while CBP KD in NDC neurons resulted in a smaller upregulation of BACE1, and EP300 KD in NDC neurons did not change BACE1 expression (Additional file 2: Fig. S12). These results corroborate our observation that ABCA7, which inhibits BACE1 transcription, decreases upon EP300/CBP KD in APP^Dup^ but not in NDC neurons (Fig. 6C, D). Interestingly, while EP300 and CBP KD samples display overall similar expression changes and cluster together separately from control samples, CBP KD results in a higher magnitude of amyloid-reducing gene downregulation compared to EP300 KD (Fig. 6C, D). Together, we conclude that an amyloid-exacerbating transcriptional program enacted by EP300 and CBP KD in APP^Dup^ extended to both primary and secondary transcriptional regulation.

We predicted that EP300/CBP KD would increase disease promoting Aβ42 secretion, because (1) Aβ-reducing genes were downregulated in EP300/CBP KD, and (2) EP300/CBP regulated genes were enriched for genes upregulated in healthy aged human brains compared to AD brains. We thus measured, via ELISA, the amount of Aβ42 secreted into the media of APP^Dup^ and NDC day 21 neurons treated with siRNA KD. Treatment with EP300/CBP siRNA compared to control KD increased Aβ42(1–42) (Aβ42) abundance in both lines of APP^Dup^ neurons, but did not alter Aβ42 abundance in NDC neurons (Fig. 6E). As controls, there were no differences in cell viability as measured by Trypan blue staining (Fig. 6F), or in total cell count (Fig. 6G) resulting from EP300/CBP KD compared to control siRNA in any cell line. Taken together, these data provide evidence that a homeostatic genetic response to increased amyloid-beta production is induced in APP^Dup^ and that the genes involved in this response, along with other amyloid-reducing genes, are regulated by EP300 and CBP.

## Discussion

There exist disparate lines of evidence regarding the nature of epigenomic dysregulation in neurodegenerative Alzheimer’s disease. We and others have reported differential H3K27ac in human brain tissue that is dependent on the brain region surveyed: H3K27ac increases in the lateral temporal lobe [14], but decreases in the entorhinal cortex of AD patient brains [15]. We also examined the histone acetylation mark H4K16ac and discovered a decrease in the brains of AD patients [9], and studies in fly models of AD suggest a protective role of H4K16ac against AD pathology-related insults [68, 69] Additionally, in a mouse model of AD, the histone deacetylase HDAC2 increases and H4K12ac decreases [70], and an ameliorative effect was demonstrated in an AD mouse model of increasing acetyl-CoA synthetase 2 (ACSS2) [71], which generates acetyl-coA and regulates histone acetylation in rodent hippocampus to promote memory [72, 73]. These findings fuel speculation that histone deacetylase inhibitors could be potential therapeutics in Alzheimer’s disease and other neurodegenerative diseases, to overcome a disease-associated “epigenomic blockade” [70, 74].

In this study, we utilized iPSC-neurons derived from familial AD patients with an APP duplication as a model to study the functional effect of reducing the key acetyltransferases EP300 and CBP. Knockdown of the acetyltransferases reduces acetylation at H3K27, and lowers acetylation of other histone and non-histone proteins [10]. As we do not observe differences in EP300 or CBP expression between APP^Dup^ and NDC neurons (Additional file 2: Fig. S4A,B) and did not assess for gross changes in H3K27ac between APP^Dup^ and NDC neurons, we cannot determine if higher levels of H3K27ac at baseline is a fundamental characteristic of APP duplication. However, in our iPSC-neurons, EP300 or CBP knockdown reduced total H3K27ac abundance (Fig. 3D, E), which correlated with widespread downregulation of gene transcription in both APP^Dup^ and NDC neurons (Fig. 4A, B, Additional file 2: Fig. S5A, B), including important genes in Alzheimer’s disease pathways (Fig. 4F, G, Additional file 2: Fig. S5F, G, Additional file 2: Fig S6, Additional file 2: Fig. S7). Knockdown of the two enzymes resulted in broadly similar transcriptional changes (Fig. 4C), and learning and memory and neuron-related processes were affected in both EP300 and CBP knockdown (Fig. 4H, I). Previous studies of EP300/CBP in the context of AD focused primarily on the well-established roles played by these acetyltransferases in learning and memory [75]. Our study uncovers a novel link between EP300/CBP and amyloid pathology in AD.

We found that a subset of genes in the APP-Aβ pathway responsible for amyloid clearance and prevention are strongly upregulated in APP^Dup^ neurons compared to NDC neurons (Fig. 6B), suggesting activation of a homeostatic genetic response to compensate for increased abundance of APP and production of downstream Aβ species. Compared to knockdown in NDC neurons, knockdown of either EP300 or CBP in APP^Dup^ neurons resulted in stronger downregulation of genes with amyloid-reducing function (Fig. 6C, D), including the APP^Dup^-upregulated, compensatory genes mentioned above. Importantly, likely as a consequence of downregulation of these amyloid-reducing gene programs, knockdown of EP300 or CBP causes an increase in abundance of disease-associated Aβ42 secreted by APP^Dup^ neurons (Fig. 6E). Moreover, the knockdown of CBP appears to more strongly affect the amyloid reduction pathways compared to knockdown of EP300, which is especially interesting in light of our previous observation of disease-specific activation of CBP and not EP300 in postmortem AD brains [15].

Our findings reveal several pathways potentially impacted by EP300/CBP KD leading to increased toxic Aβ42. LPL is strongly expressed by neurons [76] and is involved in extracellular association and sequestration of amyloid-β. Interestingly, we discovered that LPL was among the most significantly downregulated genes in neurons with EP300/CBP KD (Fig. 4A, 4B). Consistent with this finding, a small molecule inhibiting EP300/CBP catalytic activity strongly decreases LPL expression in mouse adipocytes [77]. LPL is an amyloid-β-binding protein promoting cellular uptake and clearance of amyloid-β in astrocytes [63]. Taken together, downregulation of LPL by KD of EP300/CBP may lead to increased amyloid-β. Although glial cells are considered to be primarily responsible for uptake and clearance of amyloid-β [22, 78], our results open the possibility that LPL may have glial cell-independent effects on amyloid reduction, warranting further analysis. Decreased expression of BDNF may have contributed to the increase in Aβ in EP300/CBP KD cells. BDNF reduces Aβ production [49] by promoting α-secretase activity to the exclusion of β-secretase activity [47, 48] We find that EP300/CBP knockdown reduces BDNF expression, suggesting it could be a candidate for amyloid-targeting therapeutic approaches.

While our transcriptional and phenotypic data suggest a physiologic amyloid-reducing role of EP300/CBP, we did identify EGFR as a downstream central interactor of both EP300- and CBP-controlled gene expression (Fig. 4I, J), and showed that both EP300 and CBP knockdown reduced EGFR expression in APP^Dup^ and NDC neurons (Fig. 4K). EGFR has been implicated in mediating neurotoxicity downstream of Aβ, primarily via utilization of EGFR inhibitors, which reduce amyloid-associated inflammation and ameliorate memory resulting from introduced amyloid [44, 79–81]. Upregulation of the EGFR ligand, epidermal growth factor (EGF), reduces amyloid-related deficits without affecting levels of Aβ itself [82]. Therefore, it is likely that EGFR-mediated neurotoxicity is dependent on but does not contribute to amyloid pathology, and occurs after Aβ accumulation has already been established, falling downstream of the scope of our study. Nevertheless, these results indicate that although EP300/CBP regulate a protective amyloid-reducing pathway, they concurrently regulate EGFR, an Aβ toxicity-exacerbating interactor, underlining that there is a complex relationship between histone acetylation, gene activation, and the ultimate phenotype of Aβ pathology and neurotoxicity.

The limitations of our study reflect the limitations inherent in our model system. While an established single gene mutation, like APP duplication in the familial AD lines used here, likely leads to a larger and more consistent effect size on measurements compared to the use of sporadic AD lines, the low number of cell lines used in our study poses a valid concern for generalizability. Additionally, our model lacks astrocytes or other glial cell types, which may explain discrepancies between our study and others conducted in heterogenous brain tissue. For example, while we observed increased AD pathology upon KD of EP300/CBP in the form of Aβ42 secretion (Fig. 6E), it is possible that the exclusively neuronal makeup of our model prevented us from observing potential beneficial effects of EP300/CBP KD. such as the downregulation of noxious agent EGFR, which appears to enact its Aβ42-worsening effect through neuroinflammation and its interaction with glial cells [80, 81]. Of particular relevance, our previous study reporting a net increase in H3K27ac in AD brains was performed in whole brain tissue, which does indeed contain glial cell types. Thus, it is possible that different cell types experience different degrees of histone acetylation change; for example, a report examining histone acetylation changes in individual cell types identified oligodendrocytes as the primary cell type to experience H3K27ac peak increases associated with amyloid load [83].

Additionally, we limited the scope of our experiments to the effect of EP300 and CBP KD on H3K27ac and amyloid pathology. We did not investigate the relationship of these mechanisms to tau, though tau acetylation has been shown to contribute to tau pathology and dementia [84, 85]. Whether similar compensatory mechanisms against tau pathology progression exist in tau mutant iPSC-neuron models, and whether EP300/CBP and H3K27ac drives the expression of those homeostatic programs in those models, remain interesting potential future directions. Additionally, while we demonstrated that neither CBP nor EP300 KD impacted neuronal viability (Fig. 6F), we did not investigate in detail the potential loss of normal neuron function, an especially important consideration, given the well-established role of EP300 and CBP in learning and memory [40, 41, 86].

In summary, our findings suggest a complex mechanism of disease-associated EP300/CBP dysregulation and provide further evidence of the crucial role EP300/CBP plays in AD-related pathological processes. In contrast to postmortem brain tissue, in which functional experiments are not feasible, utilization of an iPSC-neuron in vitro model allowed us to address, via perturbation experiments, the role HATs play in AD. In particular, using iPSCs derived from familial AD patients with an APP duplication allowed us to isolate the role EP300/CBP plays in Aβ pathology. We show that, in neurons, EP300/CBP KD lowers H3K27ac, inhibits the expression of genetic programs compensating for increased Aβ load, and leads to increased amyloid-β secretion. Future strategies targeting the reduction or increase of histone acetyltransferases as a potential therapeutic mechanism should thus consider the broad role played by H3K27ac as a general activator of transcription and cell-type dependent effects.

## Materials and methods

### iPSC lines and maintenance

APP^Dup^ and NDC iPSC lines were previously characterized and reported [21] and were the generous gift of the lab of Lawrence Goldstein, University of California, San Diego (lines APPDp1.1, APPDp2.1, NDC1.1, NDC2.1). Established practices for maintenance and passaging of iPSC cell lines were followed [18, 21, 87]. Briefly, freshly passaged undifferentiated cells were plated on hESC-qualified Matrigel (Corning 354,277)-coated tissue-culture treated 6-well plates in Essential 8 media (Gibco A1517001) containing 10 uM ROCK inhibitor Y-27632 (Tocris 1254). Media without ROCK inhibitor was exchanged daily and wells were passaged at approximately 70–80% confluence by washing with DPBS and mechanically dissociating with ReLeSR (StemCell 100–0483) into cell cluster suspensions. Suspended cell clusters were frozen in Stem Cell Freezing Media (ACS-3020). Cells were routinely checked for mycoplasma contamination.

### Verification of APP copy number

Verification of APP copy number was performed by quantitative PCR (qPCR) of two exon–intron junctions of APP on genomic DNA of all cell lines, normalized to genomic β-globin (*HBB*). The following primers were used for qPCR analysis: tcttcctcccacagctcctggg (FW) and gcattagccacaccagccacca (RV) (*HBB*); ttcccacccttaggctgctggt (FW) and agccttcaccttagggttgccca (RV) (*HBB*); gccaacgagagacagcagctgg (FW) and aactcggctgcagcgagaccta (RV) (*APP*); aaccaccgtggagctccttccc (FW) and ccttgctggctcaggggactct (RV) (*APP*).

### Transfection of cell lines with NGN2 construct

Creation of doxycycline-inducible NGN2 cell lines was performed on all fAD and NDC cell lines according to a previously described method [18]. All plasmids used were the generous gift of Michael Ward. Briefly, plasmid with blasticidin resistance cassette and mApple were transfected into cells. Cells were passaged and treated with by puromycin and sorted by fluorescence-activated cell sorting (FACS) for mApple positivity after 2 passages with blasticidin to select for cells with long-term stable insertion of the transgene cassette.

### iPSC-neuron differentiation

Differentiation of iPSC-neurons was based an established protocol for single-step NGN2-mediated induction of cortical neurons with minor modifications [18]. Briefly, 80% confluent wells of undifferentiated iPSCs were mechanically dissociated to single cells with StemPro Accutase (Gibco A1110501), then plated at a density of 500 k cells/well in a Matrigel-coated 6 well-plate with ROCK inhibitor (day -1). Media was changed to Neuron Induction Media 24 h after plating (day 0) and cortical neuron differentiation was induced with doxycycline (Sigma D9891). Fresh Neuron Induction Media containing doxycycline was exchanged on day 1. On day 2, wells were washed with DPBS, mechanically dissociated to single cell suspension with accutase, and plated at a density of 1 million cells per 6-well plate (or 375 k cells per 12-well plate) in poly-L-ornithine (Sigma P3655) -coated wells in Neuron Differentiation Media with 10 uM ROCK inhibitor. Cells were treated for 24 h with 5 uM araC (Tocris 45–205-0) and media was replaced on day 3 with fresh Neuron Differentiation Media without ROCK inhibitor or araC. On day 4, media was replaced with Neuronal Media, and beginning on day 8, half-volume media changes with Neuronal Media was performed every 4 days until harvest. Media was collected for ELISA assays and cells were harvested with mechanical dissociation alone for RNA-seq and Western blotting assays. Cell viability was determined via Trypan Blue (ThermoFisher) staining and counting on a hemocytometer under a bright field microscope.

### RNAi knockdown/Transfection

siRNA duplexes against CBP, EP300, and a scrambled control were designed and synthesized by Horizon Discovery Biosciences Ltd. On Day 4, siRNA duplexes were diluted in Opt-MEM (Gibco 31,985,062) and combined in Lipofectamine RNAiMax transfection reagent (ThermoFisher 13,778,150) according to the manufacturer’s instructions. Mixture was added to Neuron Media at a final siRNA concentration of 25 nM and media exchange on day 4 was performed as described above. On day 8, transfection was repeated with an additional dose of Opti-MEM, Lipofectamine RNAiMax, and Neuronal Media, and siRNA duplex at a final concentration of 50 nM, concurrent with a half-volume media change, for a total of two RNAi KD treatments spaced 4 days apart on Day 4 and Day 8 respectively.

### RNA-Seq

Frozen cell pellets from at least two independent rounds of differentiation were suspended in TriZol reagent and homogenized with handheld tissue homogenizer and extracted as previously described [88]. Trizol-extracted RNA samples were quantified on Qubit before cDNA synthesis and library prep with NEB kit, then quantified with NEB Library Quant Kit before sequencing on Illumina NextSeq 550 platform. RNA-seq tag reads were aligned to the GRCh38/hg38 reference human genome assembly using STAR with default parameters.

### Immunofluorescence

For immunofluorescence experiments, induction of iPSCs into cortical neurons was performed as described above with the modification of plating cells on poly-L-ornithine coated glass coverslips on Day 2. At indicated neuronal maturity time points, immunofluorescence and imaging was performed as described previously [89]. Briefly, cells were fixed in 4% paraformaldehyde in PBS for 10 min at room temperature, washed in PBS, permeabilized in 0.5% Triton-X solution for 10 min, and then blocked at room temperature in 10% BSA-PBS solution. Fixed and permeabilized cells were then incubated with primary antibody in 5% BSA-PBS + 0.1% Tween 20 solution at 4 °C overnight. Incubation with Alexa Fluor-coupled secondary antibody was performed at room temperature for 1 h without shaking and washed in PBS. Slides were mounted in VECTASHIELD Antifade mounting medium with DAPI (Vector Laboratories H-1200–10) and imaged on a Nikon Eclipse Ti2. Images were analyzed in Fiji.

### Western blotting

Frozen cell pellets from three independent rounds of differentiation were lysed in Tris buffered saline (TBS) containing 1% NP-40 and 1 mM freshly prepared MgCl_2_ and 1:100 Halt Protease inhibitor cocktail (ThermoFisher 78,430). SDS was added to a final concentration of 1% and samples were boiled at 95 °C for 10 min. Samples were spun at top speed in a tabletop centrifuge and quantified by Qubit and BCA protein quantification assay before loading in a NuPage 4–12% Bis–Tris gel (ThermoFisher NP0322BOX) and subjected to electrophoresis. Samples were transferred onto a 0.2 μm PVDF membrane and blocked for one hour at room temperature in 5% milk-TBS solution containing 1% Tween (TBST). Primary antibodies were added in 5% milk-TBST solution overnight, followed by three washes in TBST. Secondary antibody conjugated to HRP was performed in 5% TBST for 2 h before 3 washes in TBST. Membrane was imaged by Fujifilm LAS-4000 imager. Quantification of blot intensity was performed in ImageStudioLite v5.2.5. Ratio of H3K27ac intensity to H3 intensity was taken and normalized to CTRL RNAi H3K27ac/H3 intensity for each batch to control for batch variation in intensity. Bar plot and statistical testing (Tukey’s multiple comparison test) were prepared in Prism 10 software v10.0.0.

### Data analysis

For all analyses, raw counts files were loaded into R and quality control metrics of total read counts > 7500 for each sample and > 5 read counts in at least 3 samples were applied. For RNAi treatment vs CTRL RNAi comparisons, design =  ~ treatment + genotype + batch was used to model the effect of treatment and genotype on transcription, with comparisons being specified between RNAi treatment and CTRL RNAi downstream of linear model building, and a log2 fold-change cutoff of > 0.50 was chosen. For APP^Dup^ vs NDC comparisons, design =  ~ genotype + batch was used to model the effect of genotype on transcription, the same adj.-p-value cutoff of 0.005 was chosen, but a relatively stringent log2 fold-change cutoff of > 0.75 was chosen to adjust for the large number of identified DEGs. DEG identification and PCA plot generation from the default parameter of N = top 500 differentially expressed genes in each comparison was performed using the DESeq2 R package version 1.38.3 [90]. Gene expression was normalized for library size and dispersion through the median ratio method provided through the estimateSizeFactors function of DESeq2 and corrected for both batch and donor variation through use of the limma package version 3.54.2 [91]. Visualization of KEGG AD pathway involvement of gene sets was generated using the pathview package v1.38.0 [92]. Tissue enrichment analysis was performed using the TissueEnrich package v1.18.0 [93]. Differentially regulated genes were visualized using the enhancedVolcano [94] package v1.16.0. Heatmaps were generated by the pheatmap package developed by Ravio Kolde (University of Tartu) v1.0.12. Dendrograms drawn and arranged using the Ward.D agglomeration method and dendrogram distances were determined by Pearson correlation distance within the pheatmap package. Differentially expressed genes between iPSC and differentiated neurons were analyzed using ConsensusPathDB Release 35 (05.06.2021) [28]. Gene expression enrichment was calculated using the fGSEA [95] R package v1.24.0 based on the Gene Set Enrichment Analysis (GSEA) algorithm [96, 97]. ClusterProfiler [39] v4.6.2 was used to perform Gene Ontology analysis of differentially expressed genes in EP300/CBP KD. Box plots were generated using ggplot2 R package v3.4.3. All analyses described above were performed with a random seed of 865 for reproducibility. The esyN v2.1 Graphs tool [42] was used to draw gene network plots, relying on BioGRID v4.4.225 and using high- and low-throughput genetic (not physical) interactions. For all analysis above, R v3.16 was employed. Additionally, Python v3.10.8 was used to process the Nat Genet 2020 Nativio et al. RNA-seq and chIP-seq data and create the boxplot in 5F, filtering out genes without H3K27ac peaks and without gene expression measurements. Where multiple peaks targeted a gene, the peak with the largest chIP-seq alteration score (|log2(H3K27ac AD/Old)|) was chosen as the representative peak for that gene, lending its score for the boxplot. The background gene list was chosen to include none of the genes in any of the CBP or EP300 KD differential gene lists, randomly sampling from all other genes in the filtered list to produce a new list with a number of genes in the largest such DEGs list (the random sample seed for this analysis was set to 100 for reproducibility).

#### Statistics

Statistics for Aβ42 ELISA, NeuN positivity, cell count, and viability were performed within GraphPad Prism 10 software. All data are presented as mean + s.e.m. One-way analysis of variance (ANOVA or one-tailed Student’s *t-*tests were performed unless otherwise noted. Statistical significance was set to *p* < 0.05 for all experiments except for DESeq analyses of in vitro neuron RNA-seq data, for which significance threshold was set more stringently, *p*-adj. < 0.005. Statistics for analyses of high-throughput data were performed within R with the ggplotstats (https://indrajeetpatil.github.io/ggstatsplot/) and ggsignif v0.6.4 [99] R packages. Hypergeometric tests for overlap between gene sets were performed using the SuperExactTest v0.12.0 R package [100], with the universe of potential overlapped genes set to the intersection of genes with mapped reads in each experiment performed. For Fig. 5F, Additional file 2: Fig. S8B, C, permutation test was performed with the coin library v1.4.2 in R.

### Supplementary Information

**Additional file 1**: **Supplementary Table 1**. APPDup vs NDC Expression of Neuronal, iPSC, and Glial Markers in Neurons. **Supplementary Table 2**. APPDup Neurons, Neuronal markers, EP300 KD vs CTRL. **Supplementary Table 3**. APPDup Neurons, Neuronal markers, CBP vs CTRL. **Supplementary Table 4**. NDC Neurons, Neuronal markers, EP300 KD vs CTRL. **Supplementary Table 5**. NDC Neurons, Neuronal markers, CBP KD vs CTRL.

**Additional file 2**: **Supplementary Figure 1**. APP^Dup^ and NDC neurons exhibit robust excitatory cortical neuron identity after single-step NGN2 induction. **A**) Quantitative PCR (qPCR) of two intron-exon junctions in APP^Dup^ locus performed on genomic DNA isolated from from APP^Dup^ and NDC iPSCs, normalized to genomic beta-globin levels. **B**) Schematic of general workflow of ngn2-inducible iPSC line development. **C**,**D**) Representative immunofluorescence staining of APP^Dup^ (**C**) and NDC (**D**) neurons for DAPI and neuronal markers Synapsin1, beta-III tubulin, and PSD95. **E**, **F**) Differentiated APP^Dup^ and NDC neurons express genes associated with cerebral cortical tissue. Cerebral cortex-associated genes are the most highly enriched gene set expressed in differentiated neurons compared to iPSCs, ranked by both fold-change (**C**) and significance (**D**). **Supplementary Figure 2**. Transcriptional differences between APP^Dup^ and NDC iPSCs. **A**) Volcano plot showing baseline gene expression differences between APP^Dup^ and NDC iPSCs. **B**) Scatterplot showing correlation between transcriptional gene expression fold-change between APP^Dup^ vs NDC neurons and APP^Dup^ vs NDC iPSCs (r_Pearson_ = 0.14, p = 1.73e-85). **C**, **D**) Bubble plots depicting top 25 terms identified by ConsensusPathDB as overrepresented in NDC (**C**) and APP^Dup^ (**D**) iPSCs. **Supplementary Figure 3**. AD Pathway Gene Expression is Affected by APP^Dup^ duplication in neurons. KEGG pathway view of AD-related differential gene expression pattern between APP^Dup^ and NDC neurons generated in Pathview. Genes are color-coded based on log fold-change in expression irrespective of significance; genes expressed highest in APP^Dup^ are coded in red, and genes expressed highest in NDC are coded in blue. **Supplementary Figure 4**. EP300 and CBP expression does not significantly differ between APP^Dup^ and NDC neurons. Neither CBP (**A**) nor EP300 (**B**) display significantly different expression between APP^Dup^ and NDC neurons. Normalized read counts obtained through RNAseq. **Supplementary Figure 5**. Transcriptional effects of EP300/CBP KD in NDC neurons. **A**, **B**) EP300/CBP KD results in widespread transcriptional downregulation in NDC neurons. Volcano plot of gene expression changes between EP300 KD vs CTRL siRNA (**A**) and CBP KD vs CTRL siRNA (**B**) in NDC neurons. **C**) EP300 and CBP KD evoke generally similar transcriptional responses. Scatterplot depicting relationship of gene transcriptional changes between EP300 KD and CBP KD (r_Pearson_ = 0.62, p = 0.00) in NDC neurons. **D**, **E**) Venn diagram showing overlaps of gene expression changes between P300 and CBP KD in NDC neurons. Jaccard index of 453/1411, p < 0.05, hypergeometric test, calculated for overlaps between downregulated genes (**D**). Jaccard index of 79/737, p = 0.05, hypergeometric test, calculated for overlaps between upregulated genes (**E**). **F**, **G**) KEGG-identified AD-associated genes are positively transcriptionally controlled by EP300 (F) and CBP (**G**) in NDC neurons. Gene set enrichment analysis (GSEA) plot depicting placement of KEGG AD genes in transcriptome ranked from highest expressed in CTRL RNAi to highest expressed in EP300 (**F**) and CBP (**G**) KD in NDC neurons. **Supplementary Figure 6**. A significant overlap exists between genes upregulated in APP^Dup^ neurons compared to NDC neurons and HAT KD downregulated genes. **A**) KEGG-identified AD-associated genes are not significantly enriched in APP^Dup^ neurons compared to NDC neurons. **B**) A significant overlap is observed between the set of genes upregulated in APP^Dup^ neurons compared to NDC neurons and the set of genes downregulated by EP300 KD in APP^Dup^ neurons (Jaccard index = 62/1687, p = 3.86e–26, the set of genes upregulated in APP^Dup^ neurons compared to NDC neurons and the set of genes downregulated by CBP KD in APP^Dup^ neurons (Jaccard index = 57/1414, p = 1.74e-26),and between all three sets of genes (Jaccard index = 37/2263, p = 1.29e–54), statistics calculated via hypergeometric test for overlaps between gene sets using the SuperExactTest R package. **Supplementary Figure 7**. EP300 KD Results in Widespread Downregulation of AD Pathway genes. KEGG pathway view of AD-related differential gene expression pattern between and EP300 KD and CTRL RNAi in APP^Dup^ neurons generated in Pathview. Genes are color-coded based on log fold-change in expression irrespective of significance; genes expressed higher in KD are coded red, genes lowest in KD are coded blue. **Supplementary Figure 8**. CBP KD Results in Widespread Downregulation of AD Pathway genes. KEGG pathway view of AD-related differential gene expression pattern between and CBP KD and CTRL RNAi in APP^Dup^ neurons generated in Pathview. Genes are color-coded based on log fold-change in expression irrespective of significance; genes expressed higher in KD are coded red, genes lowest in KD are coded blue. **Supplementary Figure 9**. Cell compartment gene ontology analysis of EP300/CBP KD in APP^Dup^ and NDC neurons and esyN analysis of EP300/CBP KD in NDC neurons. **A**) Bubble plot depicting gene ontology (GO) terms identified by ClusterProfiler as significantly enriched in the set of genes downregulated in both EP300 and CBP KD in NDC neurons. Neuron-related terms are asterisked. **B**, **C**) Bubble plots representing top Gene Ontology (GO) terms identified as enriched in genes downregulated in both EP300 and CBP KD in APP^Dup^ neurons (**B**), and in genes downregulated in both EP300 and CBP KD in NDC neurons (**C**) Neuron-related terms are asterisked. **D**, **E**) EGFR is a central interactor of genes significantly downregulated by P300 and CBP KD in NDC neurons. BioGRID-identified genetic interactions depicted by EasyNetworks (esyN) of genes significantly downregulated by EP300 (**D**) and CBP (**E**) KD. **Supplementary Figure 10**. The overlap between EP300/CBP KD and Nativio et al. 2018 “Old” genes contain many neuron-related genes. A. Significant overlap was observed between NDC EP300/CBP KD-Downregulated genes and Old-associated genes (Jaccard index = 80/2226, p =6.65e-06), but not between and NDC EP300/CBP KD-downregulated genes and AD-associated genes (Jaccard index = 40/2142, p = 0.75). Hypergeometric statistical testing was performed using the SuperExactTest R package. **B**,**C**. Bubble plots depicting top terms identified by ConsensusPathDB in the set of genes overlapping between “Old” upregulated genes identified in Nativio et al. 2018 and EP300/CBP KD-downregulated genes in APP^Dup^ (**B**) and NDC (**C**) neurons. 120 genes are represented in (**B**) and 80 genes are represented in (**C**). **Supplementary Figure 11**. EP300 and CBP-regulated genes are subject to altered H3K27ac-defined enhancer regulation in AD and Old postmortem brain tissue. **A**) EP300- and CBP-regulated genes in NDC neurons are significantly associated with either AD-specific or Old-specific H3K27ac enrichment in human postmortem tissue, when contrasted with a control group of HAT-insensitive background genes that have nearby H3K27ac enrichment in the same brains. Statistical significance calculated using permutation test. **B**) Genes sensitive to HAT KD have more H3K27ac peaks/enhancers in human postmortem brain than expected by chance. Total number of peaks in both AD and Old samples per gene was counted and averaged for the sets of genes regulated by each enzyme in both APP^Dup^ and NDC neurons. Statistical significance calculated using permutation test. **Supplementary Figure 12**. EP300 KD and CBP KD Effect on BACE1 expression in APP^Dup^ and NDC neurons. EP300/CBP KD results in markedly increased BACE1 expression in APP^Dup^ neurons, but results in a smaller increase (CBP) or no significant change (EP300) upon KD in NDC neurons. Normalized read counts obtained through RNAseq.

## Data Availability

All RNASeq count file data generated or analysed during this study are included in this published article [and its supplementary information files]. Additional datasets available from the corresponding author on reasonable request. Raw data is available at GEO (Gene Expression Omnibus) Accession GSE250567.
