# Supplementary material for: Histone acetylation in an Alzheimer’s disease cell model promotes homeostatic amyloid-reducing pathways
Source: Acta Neuropathol Commun. 2024 Jan 2;12:3. doi: 10.1186/s40478-023-01696-6 (PMC10759377; doi:10.1186/s40478-023-01696-6)
Supplement: Supplementary file 2 — Additional file 2: Supplementary Figure 1. APPDup and NDC neurons exhibit robust excitatory cortical neuron identity after single-step NGN2 induction. A) Quantitative PCR (qPCR) of two intron-exon junctions in APPDup locus performed on genomic DNA isolated from from APPDup and NDC iPSCs, normalized to genomic beta-globin levels. B) Schematic of general workflow of ngn2-inducible iPSC line development. C,D) Representative immunofluorescence staining of APPDup (C) and NDC (D) neurons for DAPI and neuronal markers Synapsin1, beta-III tubulin, and PSD95. E, F) Differentiated APPDup and NDC neurons express genes associated with cerebral cortical tissue. Cerebral cortex-associated genes are the most highly enriched gene set expressed in differentiated neurons compared to iPSCs, ranked by both fold-change (C) and significance (D). Supplementary Figure 2. Transcriptional differences between APPDup and NDC iPSCs. A) Volcano plot showing baseline gene expression differences between APPDup and NDC iPSCs. B) Scatterplot showing correlation between transcriptional gene expression fold-change between APPDup vs NDC neurons and APPDup vs NDC iPSCs (rPearson = 0.14, p = 1.73e-85). C, D) Bubble plots depicting top 25 terms identified by ConsensusPathDB as overrepresented in NDC (C) and APPDup (D) iPSCs. Supplementary Figure 3. AD Pathway Gene Expression is Affected by APPDup duplication in neurons. KEGG pathway view of AD-related differential gene expression pattern between APPDup and NDC neurons generated in Pathview. Genes are color-coded based on log fold-change in expression irrespective of significance; genes expressed highest in APPDup are coded in red, and genes expressed highest in NDC are coded in blue. Supplementary Figure 4. EP300 and CBP expression does not significantly differ between APPDup and NDC neurons. Neither CBP (A) nor EP300 (B) display significantly different expression between APPDup and NDC neurons. Normalized read counts obtained through RNAseq. Supplementar [file 40478_2023_1696_MOESM2_ESM.pdf]

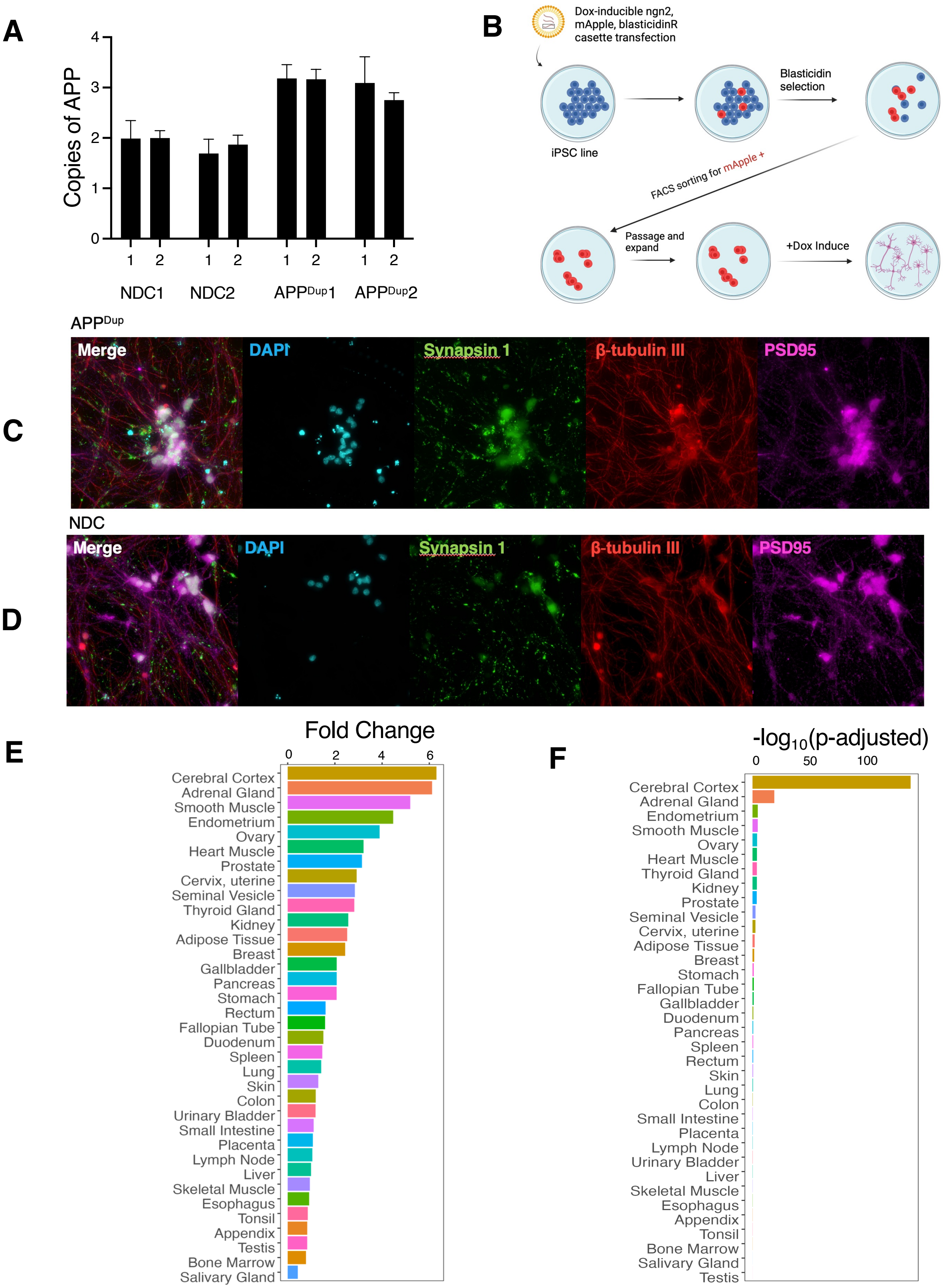

Supplementary Figure 1. APP^{Dup} and NDC neurons exhibit robust excitatory cortical neuron identity after single-step NGN2 induction. A) Quantitative PCR (qPCR) of two intron-exon junctions in *APP* locus performed on genomic DNA isolated from from APP^{Dup} and NDC iPSCs, normalized to genomic beta-globin levels. **B)** Schematic of general workflow of ngn2-inducible iPSC line development. **C,D)** Representative immunofluorescence staining of APPDup (**C**) and NDC **(D)** neurons for DAPI and neuronal markers Synapsin1, beta-III tubulin, and PSD95. **E, F)** Differentiated APP^{Dup} and NDC neurons express genes associated with cerebral cortical tissue. Cerebral cortex-associated genes are the most highly enriched gene set expressed in differentiated neurons compared to iPSCs, ranked by both fold-change **(C)** and significance **(D)**.

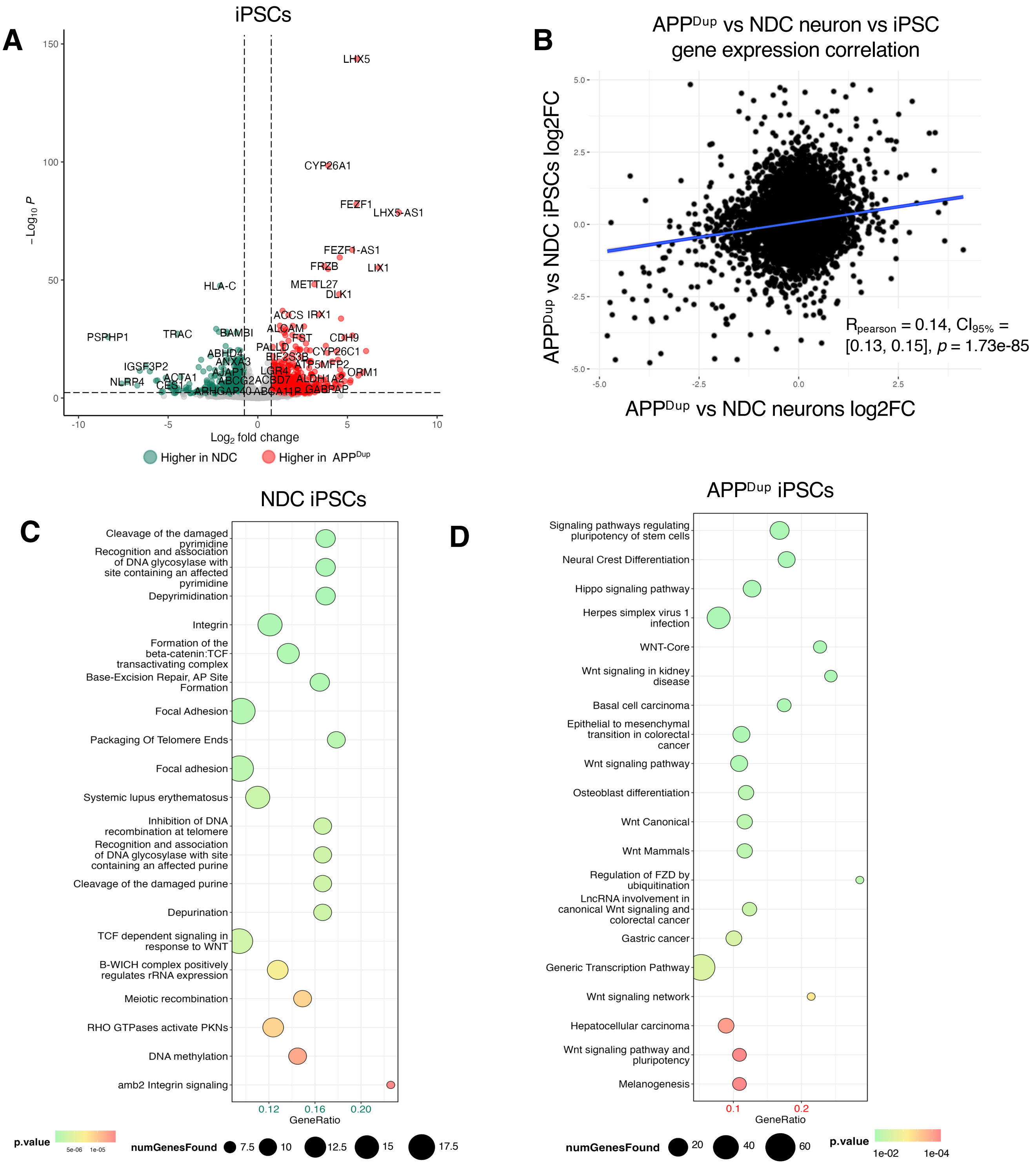

Supplementary Figure 2. Transcriptional differences between APP^{Dup} and NDC iPSCs. A) Volcano plot showing baseline gene expression differences between APP^{dup} and NDC iPSCs. **B)** Scatterplot showing correlation between transcriptional gene expression fold-change between APP^{Dup} vs NDC neurons and APP^{Dup} vs NDC iPSCs ($r_{Pearson}$ = 0.14, p = 1.73e-85). **C, D)** Bubble plots depicting top 25 terms identified by ConsensusPathDB as overrepresented in NDC **(C)** and APP^{Dup} **(D)** iPSCs.

APP^{Dup} vs NDC Neuron KEGGview

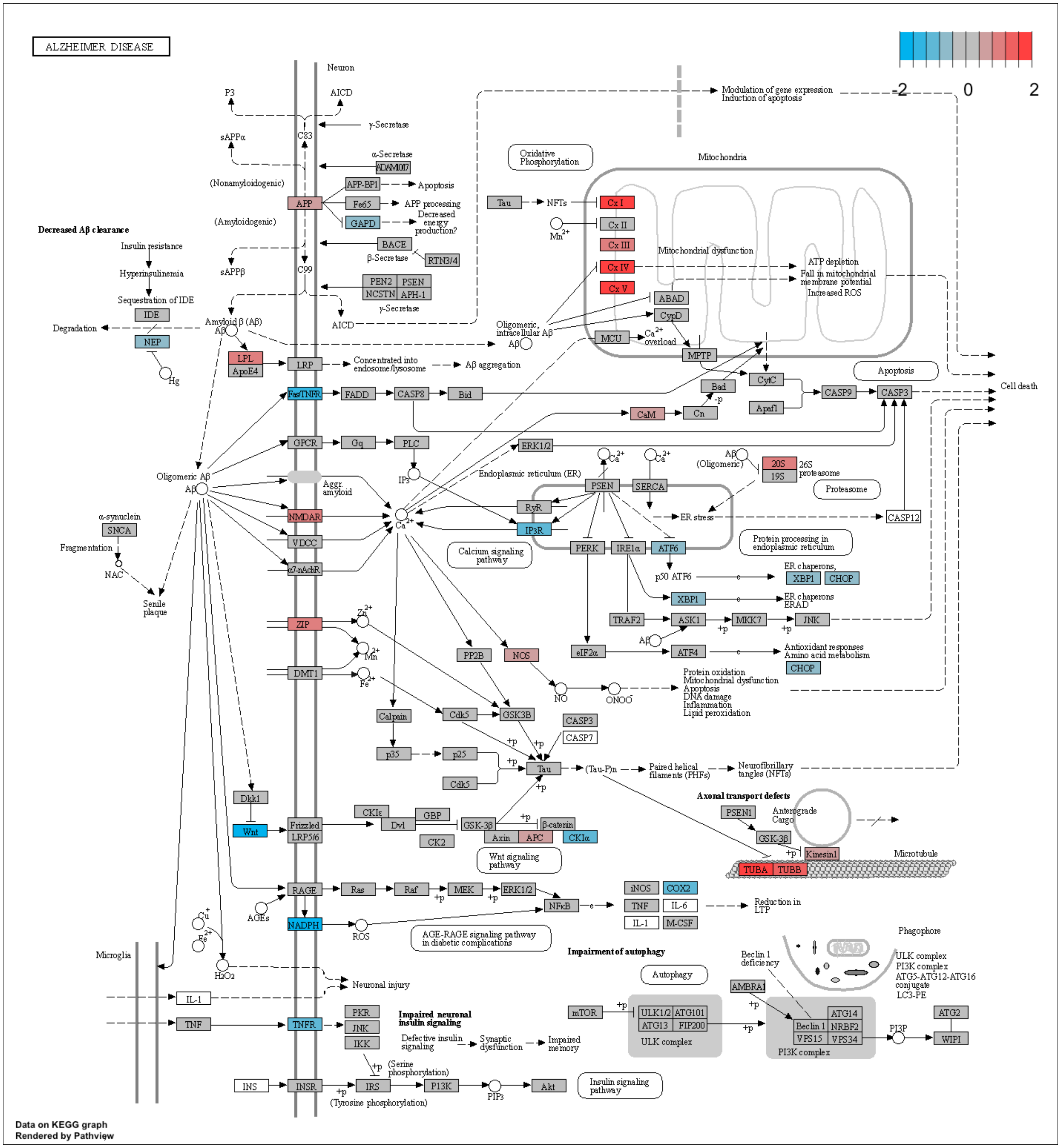

Supplementary Figure 3. AD Pathway Gene Expression is Affected by APP duplication in neurons. KEGG pathway view of AD-related differential gene expression pattern between APP^{Dup} and NDC neurons generated in Pathview. Genes are color-coded based on log2 fold-change in expression irrespective of significance; genes expressed highest in APPDup are coded in red, and genes expressed highest in NDC are coded in blue.

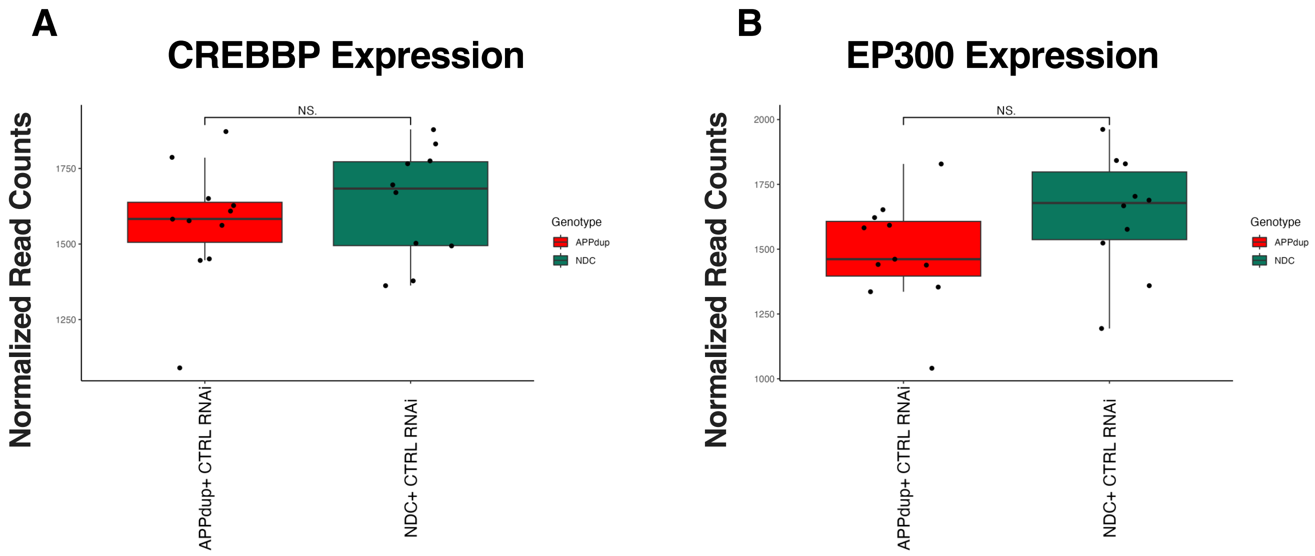

Supplementary Figure 4. EP300 and CBP expression does not significantly differ between APPDup and NDC neurons. Neither CBP (**A**) nor EP300 (**B**) display significantly different expression between APP^{Dup} and NDC neurons. Normalized read counts obtained through RNAseq.

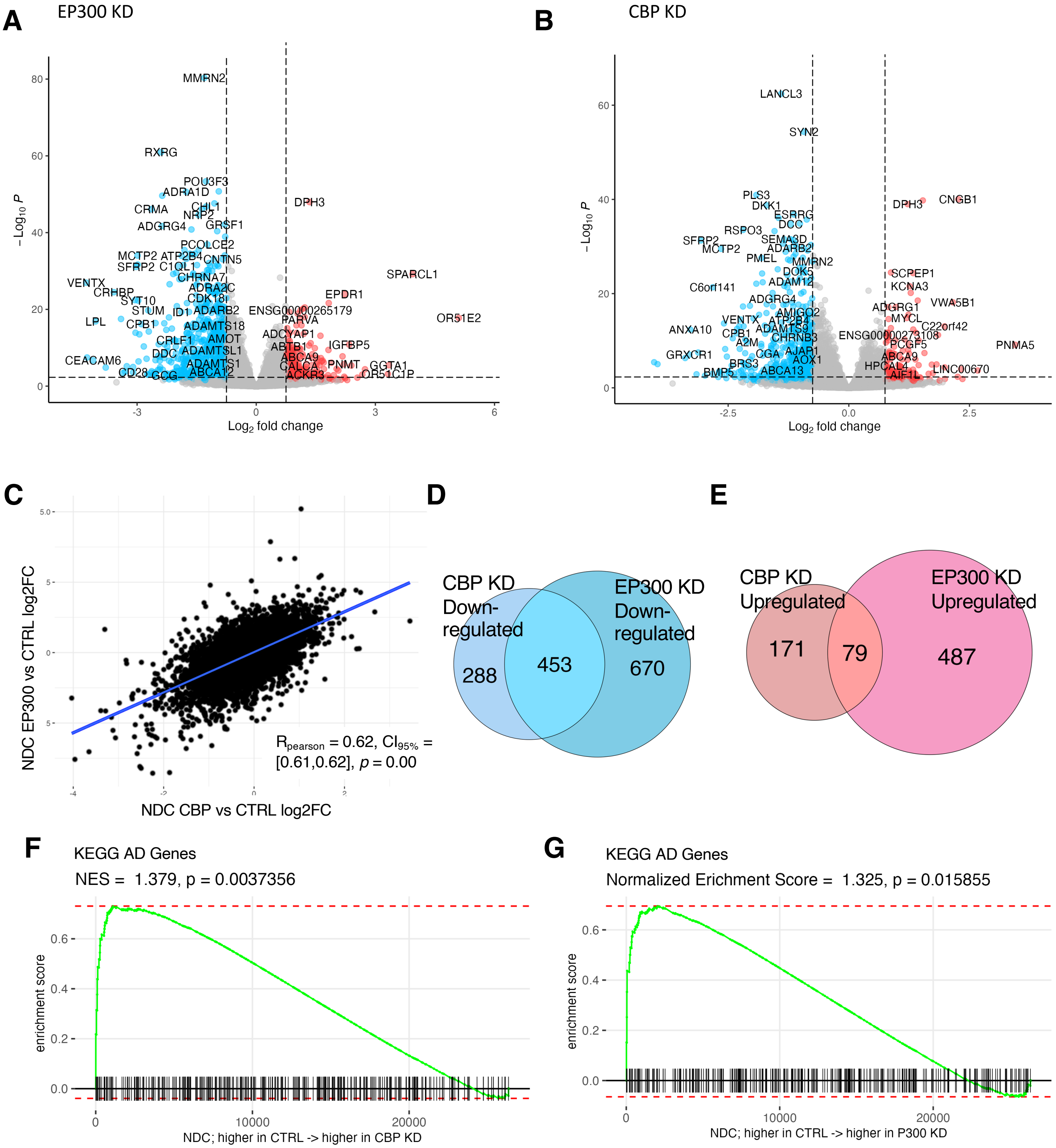

Supplementary Figure 5. Transcriptional effects of EP300/CBP KD in NDC neurons. A, B) EP300/CBP KD results in widespread transcriptional downregulation in NDC neurons. Volcano plot of gene expression changes between EP300 KD vs CTRL siRNA **(A)** and CBP KD vs CTRL siRNA **(B)** in NDC neurons. **C)** EP300 and CBP KD evoke generally similar transcriptional responses. Scatterplot depicting relationship of gene transcriptional changes between EP300 KD and CBP KD ($r_{pearson}$ = 0.62, p = 0.00) in NDC neurons. **D,E)** Venn diagram showing overlaps of gene expression changes between P300 and CBP KD in NDC neurons. Jaccard index of 453/1411, $p < 0.05$, hypergeometric test, calculated for overlaps between downregulated genes **(D).** Jaccard index of 79/737, $p = 0.05$, hypergeometric test, calculated for overlaps between upregulated genes **(E)**. **F, G)** KEGG-identified AD-associated genes are positively transcriptionally controlled by EP300 **(F)** and CBP **(G)** in NDC neurons. Gene set enrichment analysis (GSEA) plot depicting placement of KEGG AD genes in transcriptome ranked from highest expressed in CTRL RNAi to highest expressed in EP300 **(F)** and CBP **(G)** KD in NDC neurons.

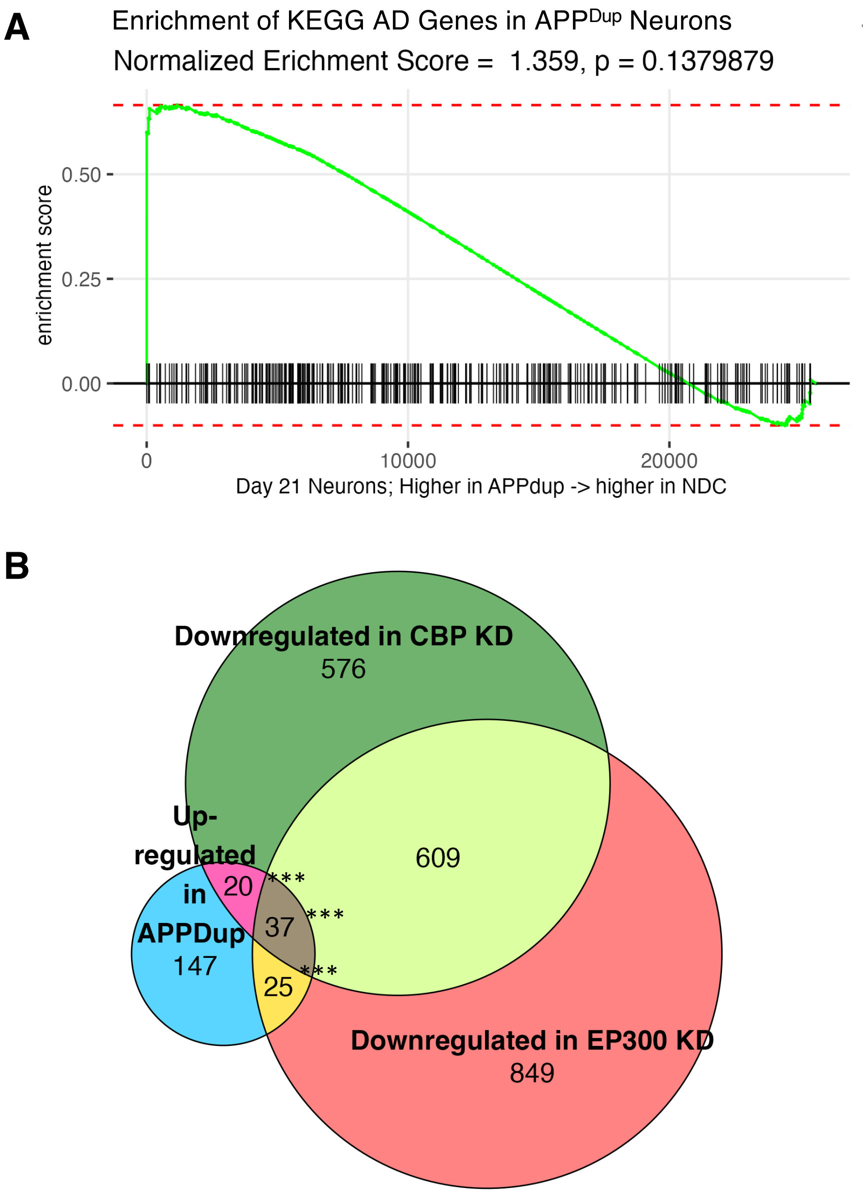

Supplementary Figure 6. A significant overlap exists between genes upregulated in APPDup neurons compared to NDC neurons and HAT KD downregulated genes. A) KEGG-identified AD-associated genes are not significantly enriched in APP^{Dup} neurons compared to NDC neurons. **B)** A significant overlap is observed between the set of genes upregulated in APP^{Dup} neurons compared to NDC neurons and the set of genes downregulated by EP300 KD in APP^{Dup} neurons (Jaccard index = 62/1687, *p* = 3.86e-26, the set of genes upregulated in APPDup neurons compared to NDC neurons and the set of genes downregulated by CBP KD in APPDup neurons (Jaccard index = 57/1414, *p* = 1.74e-26*)*,and between all three sets of genes (Jaccard index = 37/2263, *p* = 1.29e-54), statistics calculated via hypergeometric test for overlaps between gene sets using the SuperExactTest R package.

EP300 KD vs CTRL APP^{Dup} Neurons

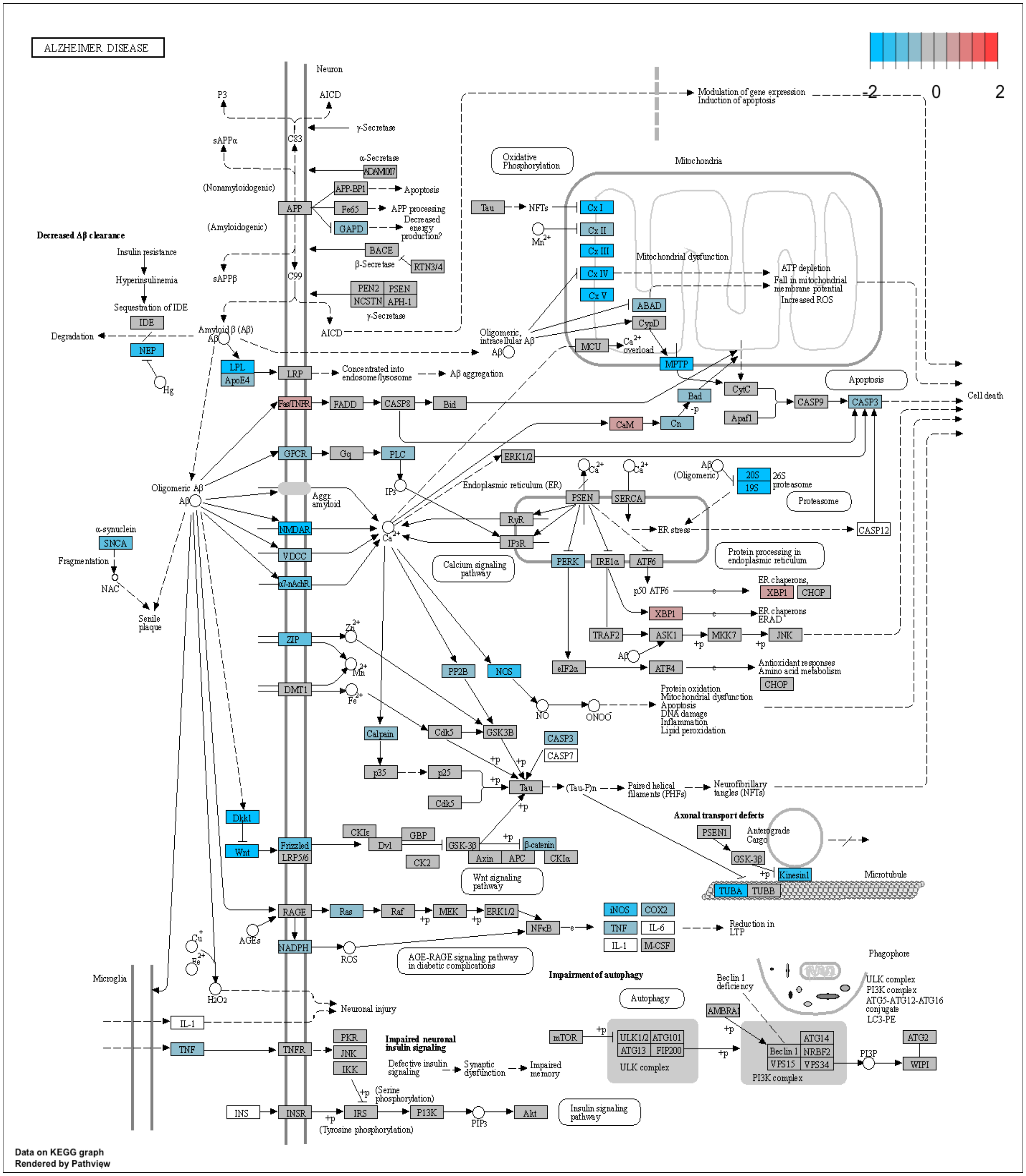

Supplementary Figure 7. EP300 KD Results in Widespread Downregulation of AD Pathway genes. KEGG pathway view of AD-related differential gene expression pattern between and EP300 KD and CTRL RNAi in APP^{Dup} neurons generated in Pathview. Genes are color-coded based on log2 fold-change in expression irrespective of significance; genes expressed higher in KD are coded red, genes lowest in KD are coded blue.

CBP KD vs CTRL APP^{Dup} Neurons

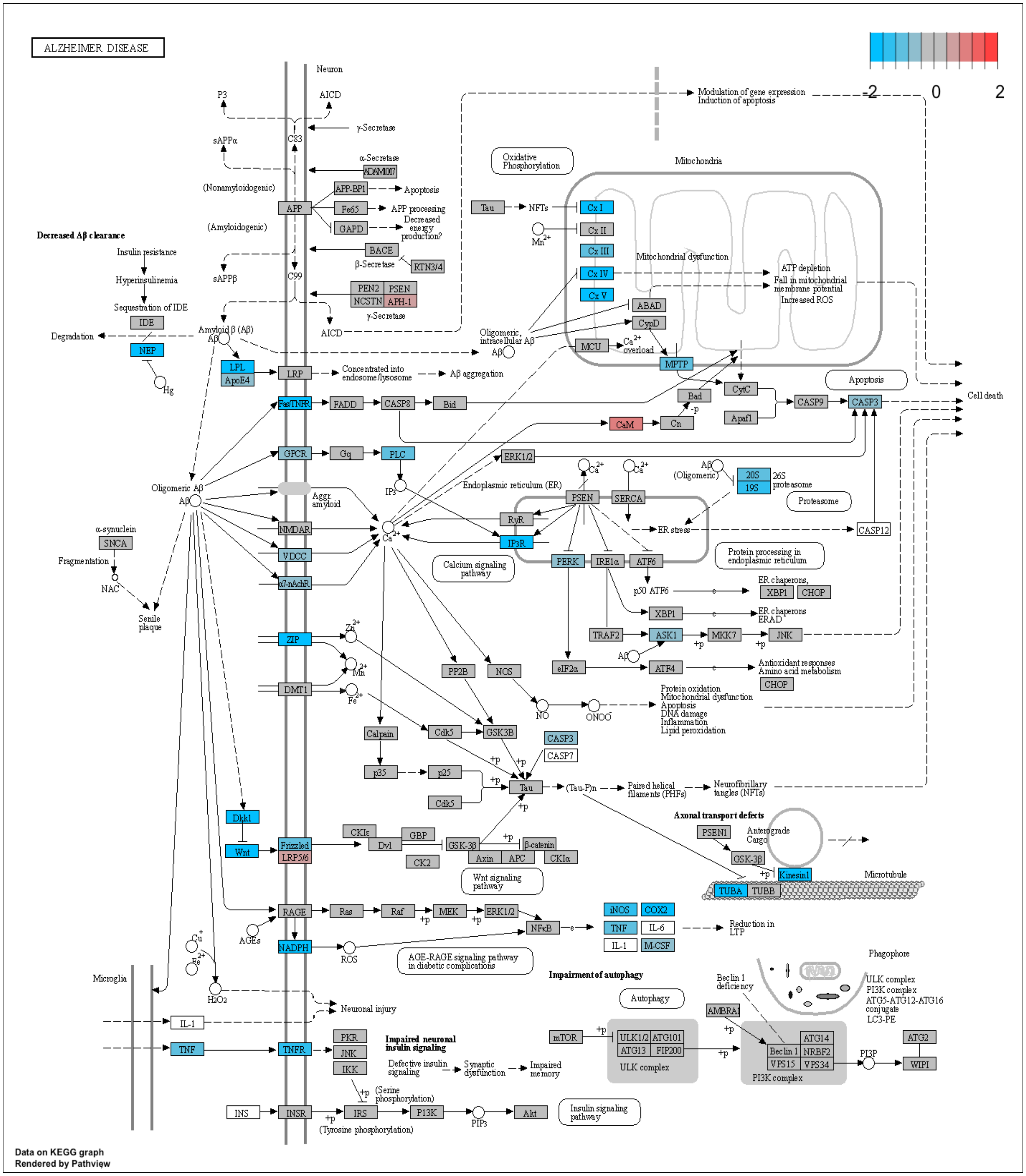

Supplementary Figure 8. CBP KD Results in Widespread Downregulation of AD Pathway genes. KEGG pathway view of AD-related differential gene expression pattern between and CBP KD and CTRL RNAi in APP^{Dup} neurons generated in Pathview. Genes are color-coded based on log2 fold-change in expression irrespective of significance; genes expressed higher in KD are coded red, genes lowest in KD are coded blue.

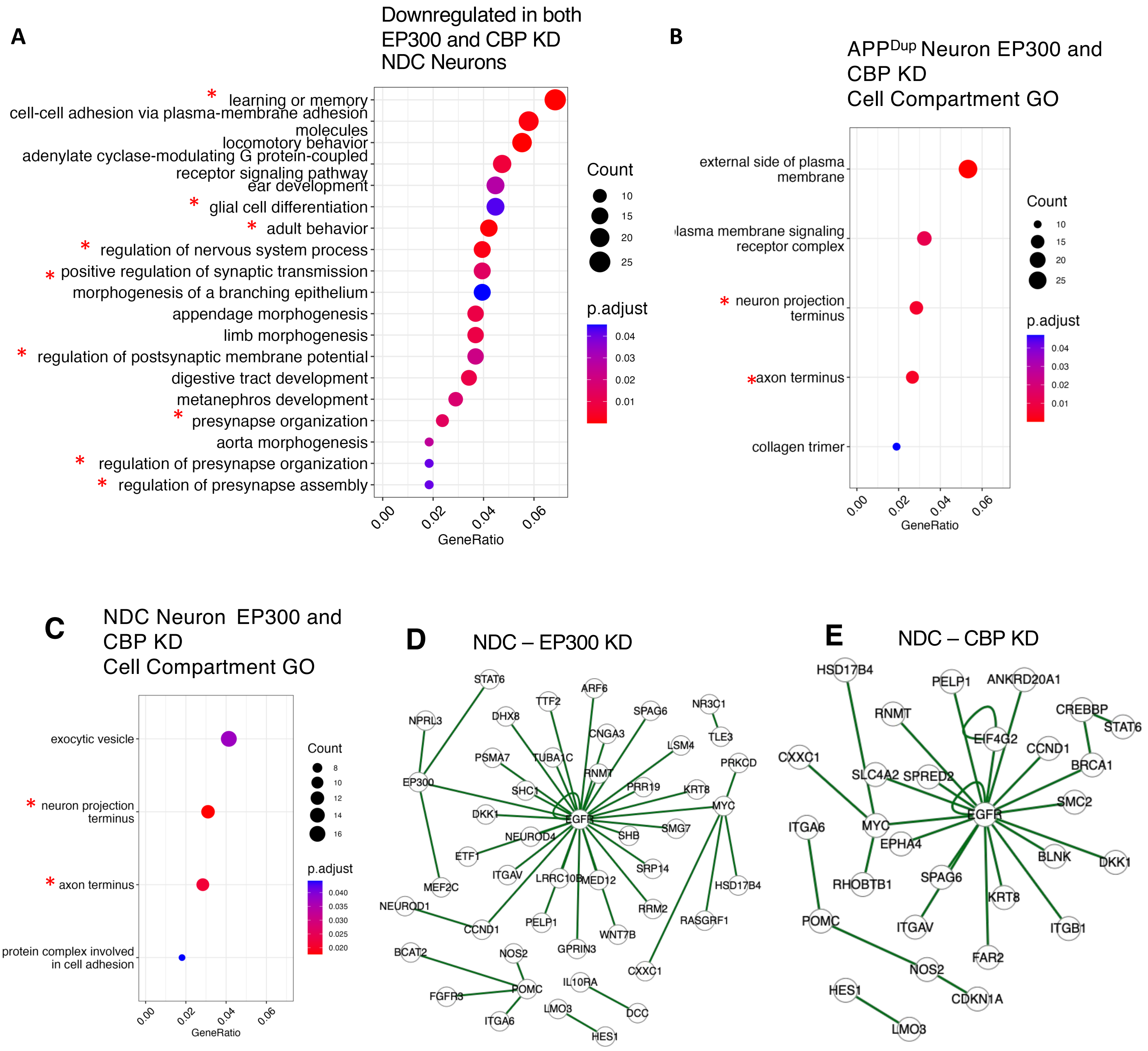

Supplementary Figure 9. Cell compartment gene ontology analysis of EP300/CBP KD in APP^{Dup} and NDC neurons and esyN analysis of EP300/CBP KD in NDC neurons. A) Bubble plot depicting gene ontology (GO) terms identified by ClusterProfiler as significantly enriched in the set of genes downregulated in both EP300 and CBP KD in NDC neurons. Neuron-related terms are asterisked. **B, C)** Bubble plots representing top Gene Ontology (GO) terms identified as enriched in genes downregulated in both EP300 and CBP KD in APP^{Dup} neurons **(B),** and in genes downregulated in both EP300 and CBP KD in NDC neurons **(C)** Neuron-related terms are asterisked. **D, E)** EGFR is a central interactor of genes significantly downregulated by P300 and CBP KD in NDC neurons. BioGRID-identified genetic interactions depicted by EasyNetworks (esyN) of genes significantly downregulated by EP300 **(D)** and CBP **(E)** KD.

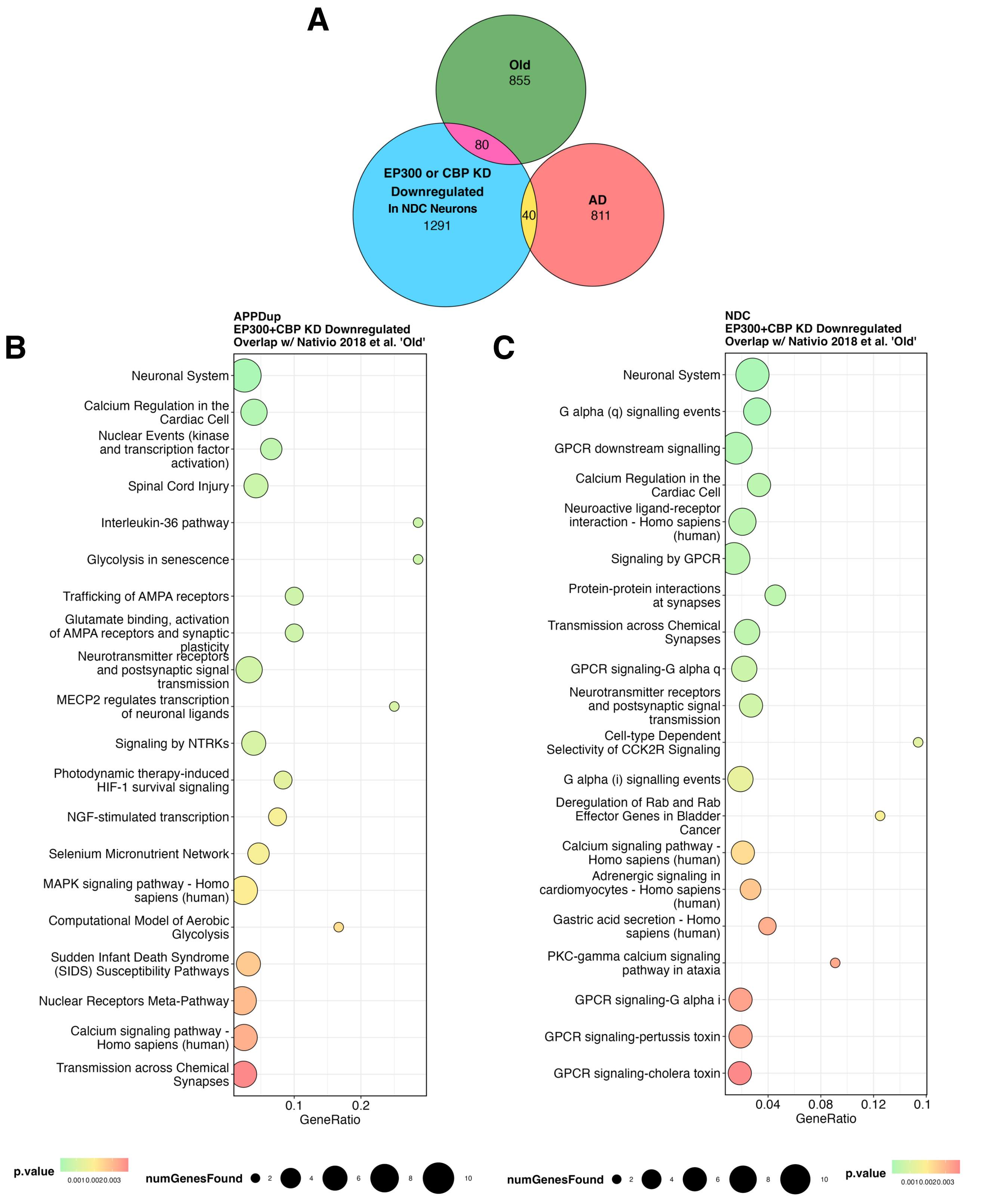

Supplementary Figure 10. The overlap between EP300/CBP KD and Nativio et al. 2018 "Old" genes contain many neuron-related genes. A. Significant overlap was observed between NDC EP300/CBP KD-Downregulated genes and Old-associated genes (Jaccard index = 80/2226, p =6.65e-06), but not between and NDC EP300/CBP KD-downregulated genes and AD-associated genes (Jaccard index = 40/2142, p = 0.75). Hypergeometric statistical testing was performed using the SuperExactTest R package. **B,C.** Bubble plots depicting top terms identified by ConsensusPathDB in the set of genes overlapping between "Old" upregulated genes identified in Nativio et al. 2018 and EP300/CBP KD-downregulated genes in APP^{Dup} (**B**) and NDC (**C**) neurons. 120 genes are represented in (**B**) and 80 genes are represented in (**C**).

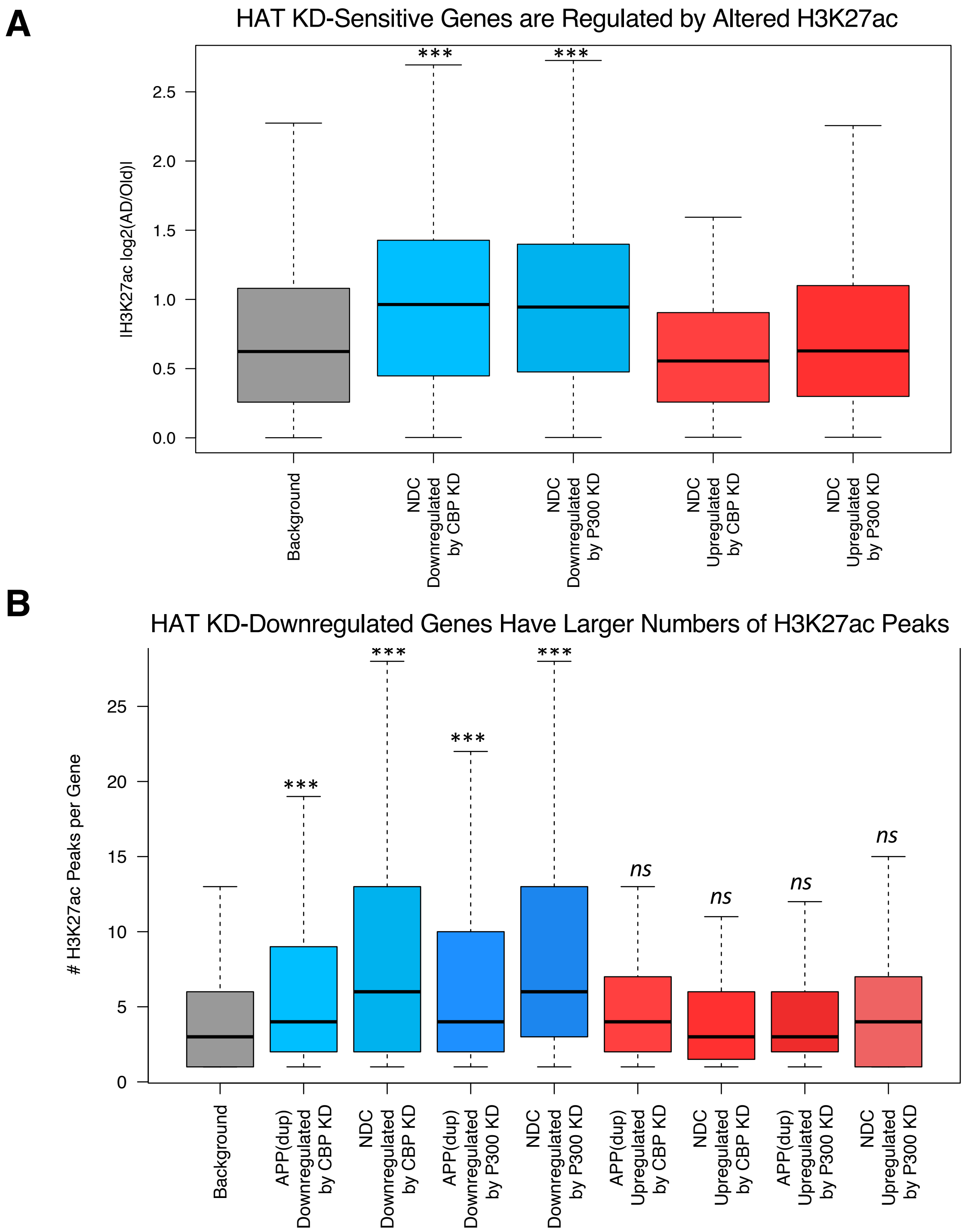

Supplementary Figure 11. EP300 and CBP-regulated genes are subject to altered H3K27ac-defined enhancer regulation in AD and Old postmortem brain tissue. **A.** EP300- and CBP-regulated genes in NDC neurons are significantly associated with either AD-specific or Old-specific H3K27ac enrichment in human postmortem tissue, when contrasted with a control group of HAT-insensitive background genes that have nearby H3K27ac enrichment in the same brains. Statistical significance calculated using permutation test. **B.** Genes sensitive to HAT KD have more H3K27ac peaks / enhancers in human postmortem brain than expected by chance. Total number of peaks in both AD and Old samples per gene was counted and averaged for the sets of genes regulated by each enzyme in both APP^{Dup} and NDC neurons. Statistical significance calculated using permutation test.

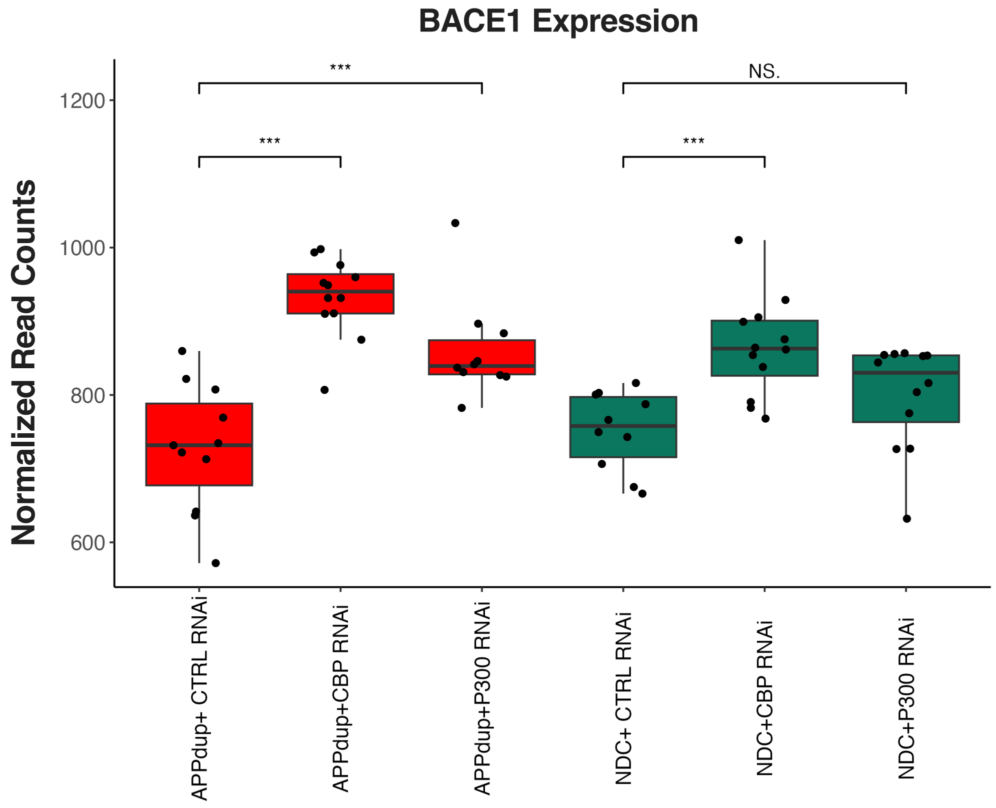

Supplementary Figure 12. EP300 KD and CBP KD Effect on BACE1 expression in APP^{Dup} and NDC neurons. EP300/CBP KD results in markedly increased BACE1 expression in APP^{Dup} neurons, but results in a smaller increase (CBP) or no significant change (EP300) upon KD in NDC neurons. Normalized read counts obtained through RNAseq.
